# Scalable design of fluidic DEMUX/MUX

**DOI:** 10.1126/sciadv.aed4468

**Published:** 2026-10-09

**Authors:** Hwayeong Jeong, Jamie Paik, Jung Kim

**Affiliations:** ^1^ École Polytechnique Fédérale de Lausanne (EPFL), Lausanne, Switzerland.; ^2^ Korea Advanced Institute of Science Technology (KAIST), Daejeon, South Korea.

## Abstract

Fluidic logic circuits offer a foundation for decentralized onboard control in soft pneumatic robots, enabling their integration into actuator networks to generate complex movements. However, existing fluidic controllers in soft robots face scalability limitations, restricting their ability to handle growing degrees of freedom across sizes while preserving compliance. Here, we present a scalable fluidic control architecture that integrates high-radix demultiplexing with time multiplexing to achieve a high input-to-output ratio and reprogrammable actuation. We incorporate a design method based on dimensionless parameters to preserve comparable valve opening and closing pressure thresholds across different physical scales, thereby maintaining consistent addressing behavior of the demultiplexer after geometric scaling. In addition, structural stretchability enables seamless integration into soft bodies, maintaining reliable control under dynamic deformation without compromising compliance. This hybrid demultiplexer/multiplexer fluidic circuit overcomes key limitations, offering functional scalability, geometric scalability, and physical integrability in a single architecture. Our approach enables autonomous, adaptable soft robots capable of complex, multifunctional motions, functioning as self-contained systems.

## INTRODUCTION

Fluidic logic circuits provide a promising framework for decentralized, onboard control of pneumatic systems (*1*–*3*). By integrating logic within actuator networks, these systems can execute complex motions using air as both the power and control medium. This approach simplifies system architectures, reduces dependency on external components, and has been widely adopted in microfluidic circuits, where fluid flow alone governs logical operations (*4*–*6*). Recently, this concept has been extended to soft robotics, exploiting the shared reliance on fluidic actuation (*7*–*13*). Conventional designs, such as Quake valve–based logic gates (*14*) and more advanced mechanically intelligent valve architectures, have enabled the creation of circuit elements including resistors, amplifiers, oscillators, and shift registers for soft robot control (*15*–*21*). However, as soft fluidic robots become more functionally capable and increase in complexity (*22*–*24*), controlling their behavior in a scalable and robust way becomes a challenge.

The challenge lies in coordinating an increasing number of degrees of freedom (DoF) across soft robotic bodies of varying scales, while preserving their inherent mechanical compliance. Expanding controllable DoF is essential for achieving diverse motion patterns and supporting increasingly sophisticated tasks. While biological organisms exhibit remarkable dexterity and adaptability in complex environments, current soft robots remain limited in both behavioral complexity and functional versatility (*25*, *26*). Bridging this gap requires control architectures with functional scalability, the ability to manage an increased number of DoF while remaining within practical system constraints. In addition to functional scalability, control systems must also support geometric scalability, the ability to adapt to different tasks, actuator types, and robot sizes without requiring extensive redesign. This adaptability is increasingly vital, as soft robots are expected to operate across a broad range of applications and scales. Equally important is the physical integration of the control system within the robot body. Embedding the controller directly into soft structures inherently subjects it to the same mechanical deformations, such as stretching, bending, and compression, that the robot experiences during operation. As a result, the controller must maintain stable control functionality under these dynamic conditions. At the same time, the presence of the controller should minimally interfere with the robot’s natural motion, preserving the overall compliance and responsiveness of the system.

A promising direction explored by researchers involves the use of fluidic demultiplexers (DEMUXs) and matrix architectures for low-level motion control in soft robotics (*14*, *27*, *28*). Fluidic DEMUXs offer input reduction by routing flow from a single source to multiple actuators, controlling up to $2^{N-1}$ outputs with *N* inputs (*14*). This approach offers a compact and scalable alternative to traditional control systems. In contrast, fluidic matrices, inspired by dot-matrix displays, enable the generation of more complex actuation patterns through time-multiplexed activation, accumulating multiple outputs across different time frames. To facilitate physical integration of these circuits into soft robotic bodies, researchers have developed fluidic systems that are either highly miniaturized or constructed from materials that are mechanically compatible with, or identical to, those used in soft robots (*29*–*31*). In particular, material uniformity reduces interface mismatch and enables controllers to be seamlessly embedded and distributed throughout the body. In doing so, it becomes possible to design systems where both actuation and control are entirely soft and colocated, paving the way for more autonomous and fully integrated soft robots (*10*, *14*, *15*).

Despite this progress, current fluidic control strategies remain limited in their ability to address all three key challenges simultaneously: (i) low reprogrammable pattern fidelity limiting functional scalability, (ii) lack of a method for geometric scaling across robot sizes, and (iii) difficulty maintaining desired control functionality after physical integration within compliant, deformable bodies. Fluidic DEMUXs offer input efficiency but inherently allow only one output to be activated at a time, constraining multichamber coordination and limiting motion diversity. Fluidic matrices, while enabling richer pattern generation through time multiplexing, require substantially more input channels than DEMUXs, increasing connection complexity. Both approaches also fall short in terms of geometric scalability. Fluidic circuits for soft robots are often tailored to fixed size ranges, and many do not provide methods for resizing or reconfiguring for different applications. This limits their application to robots of different sizes and types. Physical integration remains problematic as well. Miniaturizing valves can reduce flow delivery capability by increasing fluidic resistance and limiting the pressure-flow capacity of the pneumatic supply, while using materials identical to soft actuators exposes control components to mechanical disturbances. These effects are amplified by the nonlinear sensitivity of flow resistance to channel geometry, making embedded fluidic systems prone to instability, blockage, and failure under deformation.

This study presents a physically integrable and geometrically scalable fluidic controller designed to enhance the functionality of soft robots through a hybrid demultiplexing and time multiplexing approach. The proposed architecture enables a large number of independently addressable output states using a limited number of external fluidic input channels, thereby converting limited input dimensionality into rich functional output complexity. By combining high-radix demultiplexing with time-multiplexing actuation, the system achieves a high input-to-output ratio and supports diverse, reprogrammable actuation patterns. To support geometric scaling, the controller incorporates a scalable design methodology aimed at maintaining comparable valve opening and closing pressure thresholds across different robot sizes, thereby preserving consistent DEMUX addressing behavior after scaling. For seamless physical integration, it features structurally stretchable components that minimize interference with robot dynamics while maintaining robustness under external mechanical disturbances. Overall, this work introduces a fluidic DEMUX/multiplexer (MUX) architecture that addresses a fundamental challenge in soft robotics by realizing the combined benefits of functional scalability, geometric adaptability, and physical integrability.

## RESULTS

### Design challenges of fully soft controller

We introduce an innovative design strategy aimed at realizing a fully soft controller that simultaneously achieves functional scalability, geometric scalability, and physical integrability. The goal is to support the control of an increasing number of DoF across soft robotic bodies of varying sizes, while enabling seamless physical integration within the robot structure (Fig. 1A). The following sections delve into the key challenges associated with each of these aspects and outline our approach to overcoming them within a fully integrated soft robotic system.

**Fig. 1.**
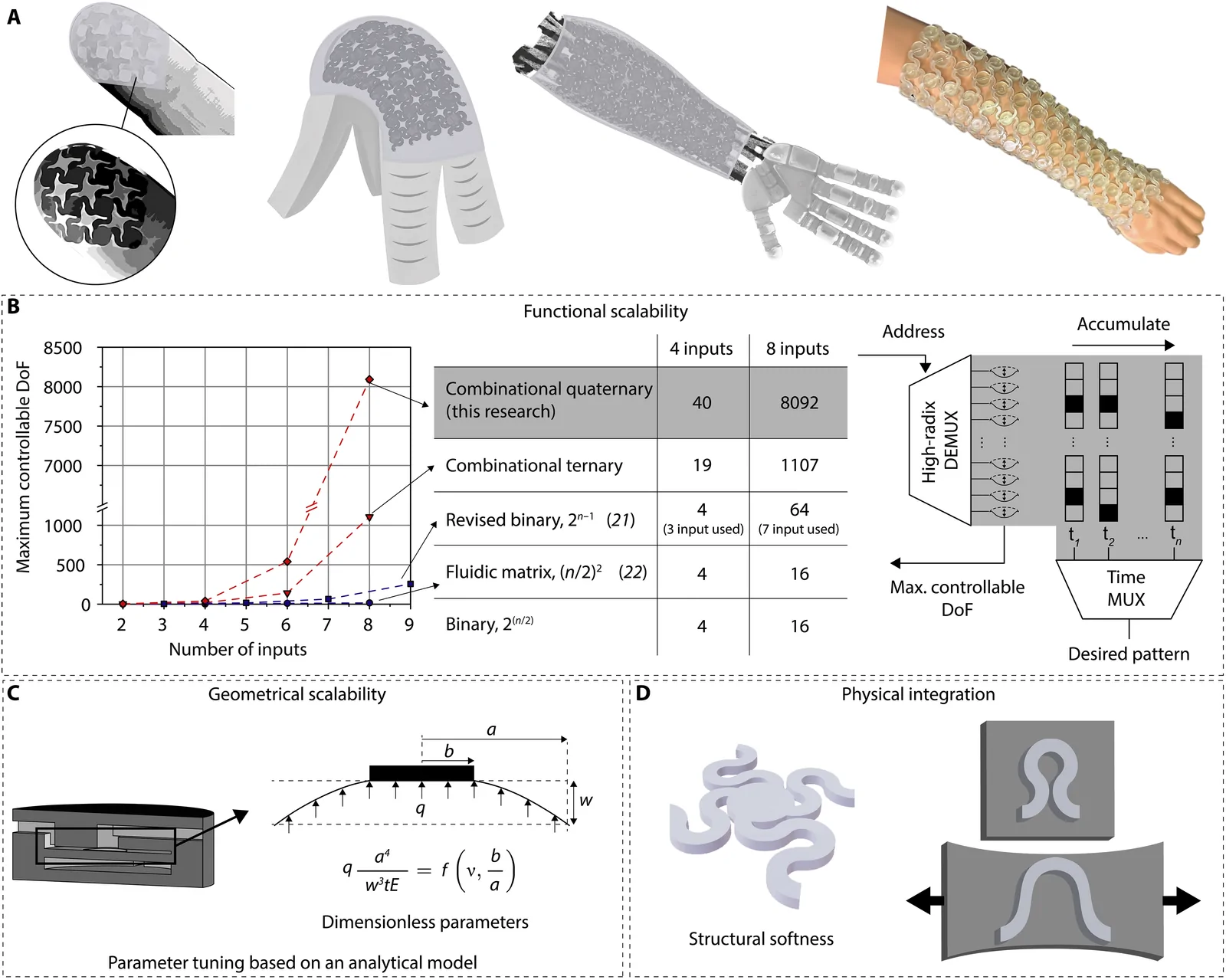
Three key design challenges in fluidic controllers for soft robots addressed in this research. (**A**) A fully integrated fluidic controller that operates across robots of varying sizes, enabling control of an increasing number of DoF. (**B**) Functional scalability via a DEMUX/MUX controller. (**C**) Geometrical scalability through parameter tuning based on an analytical model. (**D**) Physical integrability through structurally stretchable fluidic channels that maintain stable flow resistance under deformation while minimizing interference with the robot’s compliance and motion.

#### Functional scalability

A key design requirement for fluidic controllers in soft robots is functional scalability, defined here as the ability to generate an increasing diversity of actuation patterns without requiring a proportional increase in the number of external fluidic input channels. This requirement is important because external fluidic connections can directly affect the complexity, size, kinematics, and dynamics of soft robotic systems. Therefore, the controller should support diverse and reprogrammable output states during operation while using a limited set of shared fluidic input channels. For example, in applications such as catheter-based or minimally invasive soft robotic tools, as well as dense actuator arrays for shape-morphing surfaces, the number of independently routable fluidic input channels is often severely constrained by space and integration limits. Under these constraints, sharing a limited number of input channels across multiple outputs enables greater actuation pattern diversity without requiring a dedicated fluidic line for every output. Existing approaches often fall short, offering only fixed, limited patterns or requiring many input lines for pattern diversity.

Oscillator-based or fluidic computing architectures can actuate multiple DoF from a common pressure source without electromechanical components; however, they are limited to fixed actuation patterns and cannot be dynamically reprogrammed during operation (*9*, *11*, *12*, *15*, *16*). Binary fluidic DEMUXs improve input efficiency but are inherently designed to select only one output channel at a time, preventing simultaneous multichamber actuation (*14*). Fluidic matrices enable richer pattern generation through time multiplexing but require substantially more input channels, thereby increasing connection complexity (*27*).

To address these challenges, this study introduces a fluidic hybrid DEMUX/MUX controller that combines a high-radix DEMUX with time multiplexing to independently control multiple actuators and generate complex multichamber actuation patterns, while maximizing pattern diversity under a limited number of fluidic input channels (Fig. 1B). Fluidic matrices and binary DEMUXs show limited output expansion, scaling as ${\left(n/2\right)}^{2}$ and $2^{n/2}$, respectively, with n inputs. Previous research on binary DEMUXs has improved their scalability, achieving $2^{n-1}$ outputs with n inputs (*14*). However, the advantages of DEMUXs are still less notable, especially when the number of desired outputs is low. On the other hand, using ternary or quaternary logic and integrating valve closure dependency into an architecture, the system can achieve 1107 and 8092 outputs with the same 8 inputs, demonstrating enhanced scalability and versatility (*32*, *33*). Furthermore, a memory element is integrated at each output of the DEMUX to support time-multiplexed actuation, enabling the system to realize all $2^{N}$ possible activation patterns of N digital chambers, which corresponds to the number of outputs of the DEMUX. In this architecture, the valve transiently addresses each chamber, whereas the memory element preserves the resulting chamber state after actuation. Many methods can be used as a memory element; however, in this research, we used a bistable structure to achieve nonvolatile mechanical memory through snap-through stability, enabling reliable state retention and sequential pattern accumulation (*34*). Together, these features enhance functional scalability by allowing a wide range of actuation patterns while maintaining a compact input interface.

#### Geometrical scalability

Geometrical scalability is closely linked with functional scalability, as a fluidic controller should be adaptable to robots with different sizes and layouts while preserving its intended addressing behavior. This scalability enables the controller to integrate seamlessly into robots of different sizes, from compact units to large-scale systems. However, in soft robotic fluidic circuits, geometric scalability is often overlooked. Many existing controllers are limited by fixed dimensions and layouts, restricting their adaptability and use across diverse configurations. To address these challenges, this study introduces a design methodology that allows the controller geometry to be resized or reconfigured while maintaining comparable valve opening and closing pressure thresholds. From the diaphragm model, we identified dimensionless parameters that determine the pressure required to achieve a specific deformation (Fig. 1C). By keeping these parameters constant, it is possible to design valves that close at the same pressure, regardless of their size. Overall, this scalable design enhances the versatility and applicability of fluidic controllers, making them better suited for various robotic platforms and task-specific requirements.

#### Physical integrability

The final challenge in developing an onboard pneumatic controller is creating a stretchable and flexible structure. These channels must remain robust against deformation, ensuring reliable functionality under mechanical deformation, maintaining stable flow resistance during bending, stretching, compression, and other loading conditions. However, current studies either overlook the impact of deformation on controller operation when integrated into a soft body or fail to provide sufficient flow rate and pressure due to their small size (*35*–*37*). To address this challenge, we implement structural compliance, ensuring that the system remains flexible to minimize its impact on robot dynamics while maintaining robustness against deformation (Fig. 1D). The circuit material has a Young’s modulus approximately 5 to 10 times higher than that of the soft robot body material, enhancing resilience to deformation such as bending, stretching, and compression. This allows the system to endure considerable deformation while preserving stable flow resistance. Meanwhile, the channel structure, designed with high tortuosity, enables stretching through rotational and translational motion, further enhancing flexibility and adaptability.

### Functional scalability via high-radix DEMUX and time MUX integration

Here, we demonstrate functional scalability by combining two functions; increasing the input–to–controllable output ratio and maximizing the number of patterns generated by the combined activation of outputs. A DEMUX is a control architecture that routes a single input signal to one of multiple output channels based on a selection mechanism. In fluidic systems, this is typically implemented by arranging binary valves that open or close in response to control inputs, such that only one output path remains open for a given control input condition. Each output channel contains a specific combination of valves connected to the control lines (or no valve), and if any valve along an output channel is closed by the applied control input, then flow through that output channel is blocked. A high-radix DEMUX is implemented to expand the number of controllable outputs, drawing inspiration from microfluidic systems that use multistate inputs instead of binary signals (*32*). High-radix DEMUXs exploit valves with multiple discrete switching thresholds to encode more than two selection states using a single input line. This architecture enables ternary, quaternary, and higher-radix signal representations, it does not implement a specific formal many-valued logical algebra (e.g., Kleene or Łukasiewicz logic). Instead, the multivalued behavior arises from discretization of a continuous pressure input through multiple threshold valves, each of which remains intrinsically binary (open/closed). The logical radix is therefore determined by the number of distinct valve thresholds and constitutes a high-radix physical encoding scheme rather than a truth-functional many-valued logic framework. In the proposed device, valves with distinct closing pressures are arranged in series along each output channel (Fig. 2A). All output channels share a common actuation input, while each output line contains a distinct combination of valve types. As the control input is varied across discrete pressure levels, valves with closing pressures at or below the applied level are closed, thereby blocking specific paths and selectively routing flow to the output channel that contains no closed valves. This serial arrangement of multithreshold valves enables high-radix addressing and substantially increases the number of addressable outputs per input channel. As shown in Fig. 2A, our prototype can independently control 40 outputs using four quaternary inputs, greatly enhancing the input-to-output ratio. This is achieved through a combination of multistate inputs and the valve dependency of the fluidic DEMUX, where higher state inputs close the valves assigned to lower states (Fig. 2D). This valve dependency distinguishes the fluidic DEMUX from the electrical DEMUX.

**Fig. 2.**
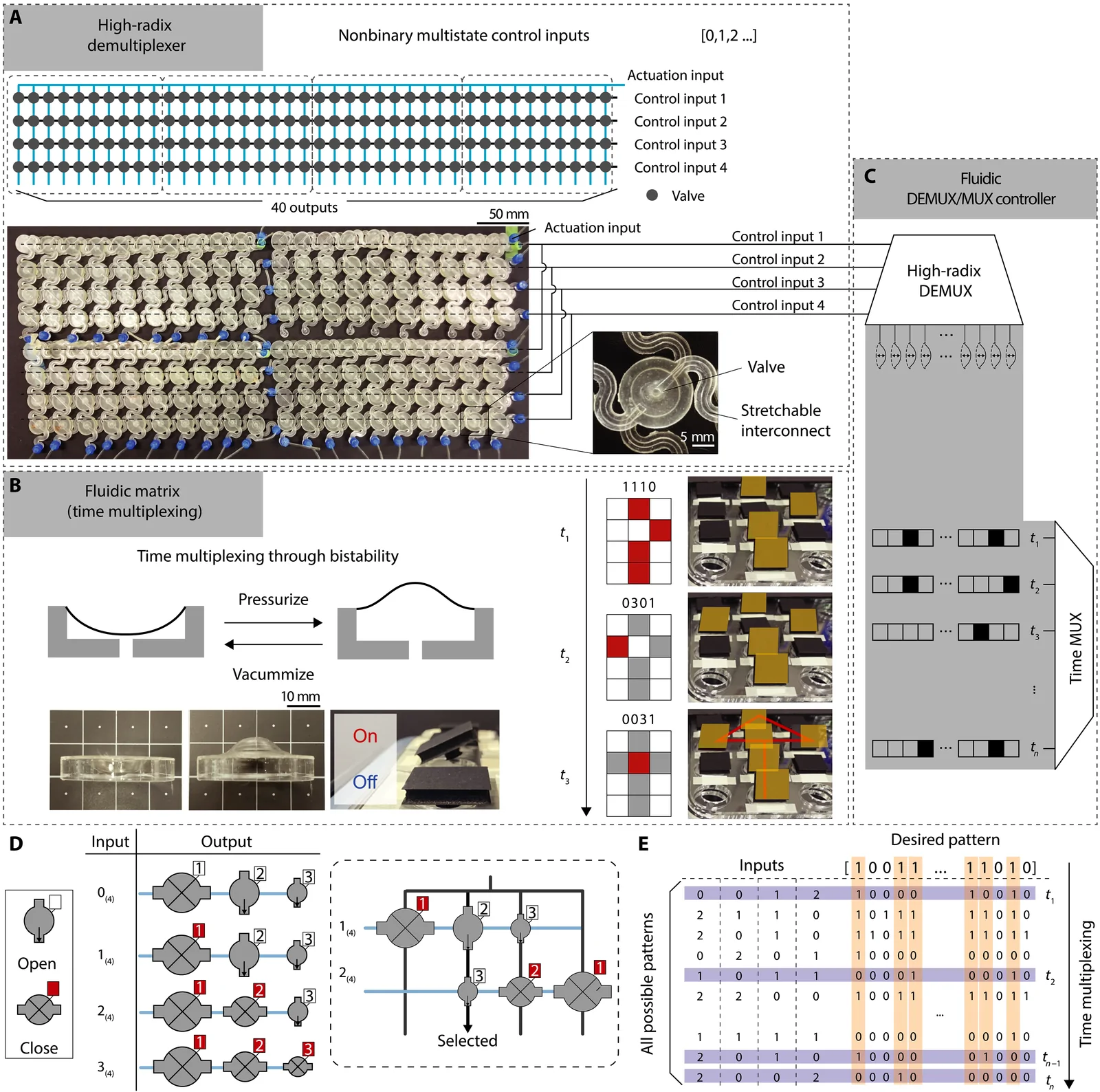
The functional scalability of hybrid fluidic DEMUX/MUX controller. (**A**) The prototype with 4 quaternary inputs, including 4 control inputs, capable of managing 40 outputs. For practical convenience during experimentation, the DEMUX was divided into four separate sections, as shown in the accompanying illustration. (**B**) Time multiplexing with bistable memory. (**C**) Integration of a high-radix DEMUX for a high input-to-output ratio and a time MUX to accumulate outputs for generating diverse patterns. (**D**) Illustration of valve dependency using a quaternary input example. (**E**) An example of selected patterns from all possible patterns to achieve the desired pattern using time multiplexing.

The total number of possible outputs in a circuit with n control inputs and m types of valves, representing m+1 radix, can be calculated using the following equation (*33*) (see note S1 for details).

Tsingle=Ca1n×Ca2n−a1×Ca3n−a1−a2×⋯×Camn−a1−a2−⋯−am−1 (1)

where

$$a_{1}+2a_{2}+\ldots +ma_{m}=\frac{m\times n}{2}$$

$$a_{1}+a_{2}+\ldots +a_{m}\leq n$$

$\left[a_{1},a_{2},\cdots a_{m}\right]$ represent the corresponding number of selections for each state.

By summing all $T_{\text{single}}$ values for each input combination, we can obtain the maximum number of addressable outputs under given conditions. DEMUX with four ternary and quaternary inputs can control 29 and 40 outputs, respectively, surpassing the capacity of current binary fluidic DEMUXs (Fig. 1B). This advantage becomes increasingly pronounced as the number of target actuators increases, since high-radix inputs reduce the required input count exponentially. While a binary architecture with 8 inputs can address maximum 128 outputs, ternary and quaternary high-radix implementations can achieve 1107 and 8092 outputs, respectively, using the same number of inputs. By increasing the information capacity of each input line, the high-radix approach enhances addressing efficiency compared to purely binary strategies.

We adopt time multiplexing to expand the functional output of a high-radix DEMUX, enabling complex actuation combinations. Similar to how light-emitting diode matrices combine multiple row activations into a unified display, we accumulate primary patterns, those generated from single input sets, to form a desired pattern (see note S2 for identifying unique primary patterns). While pneumatic switching is slow for seamless human perception, we overcome this by integrating memory at the outputs, allowing each to retain its state and support time multiplexing (Fig. 2, B and E). Combined with individual output selection, this enables the generation of all patterns that can be achieved with the given actuators (Fig. 2C and see note S3 for a detailed methodology to identify the minimum set of primary patterns needed to generate a desired pattern).

### Architectures and characterization of hybrid fluidic DEMUX/MUX

We present the controller architecture, which requires multiple valves to function at different pressures, making the following design factors essential: operational pressure range, repeatable valve response, and prevention of interference between groups. First, regarding the operational pressure range, in applying microfluidic circuit concepts to soft robots, it is essential to enhance both flow rate and pressure tolerance (*17*). Flow rate can be increased relatively easily by enlarging the system; according to the Hagen-Poiseuille equation, doubling the channel radius yields a 16-fold increase in flow rate under a constant pressure drop (*38*). In contrast, increasing the allowable actuation pressure is challenging, as it is constrained by the mechanical limits and fabrication reliability of soft materials. Fluidic valves, which operate via diaphragm-separated chambers, respond to pressure differentials and typically require control pressures exceeding actuation pressures to block flow. High-radix systems demand valves with varying closing thresholds, expanding the required control pressure range and thereby reducing the maximum usable actuation pressure. To address this, we integrated a geometric force amplification mechanism that allows the valve to remain closed under higher actuation pressure using a lower control pressure. This design keeps control pressures within or below actuation levels, improving system reliability and simplifying multistate input implementation.

We modified the plunge-type valve to incorporate a geometric force amplification mechanism, enabling valve closure under lower control pressures even when opposing higher actuation pressures (Fig. 3A). Upon application of control pressure to the control chamber (blue region), the diaphragm deflects and contacts the upper surface, sealing the central hole and closing the valve. Once the valve is closed, actuation pressure is applied to the actuation chamber, resulting in pressurization of the input channel line (red region). In this design, when the diaphragm contacts the hole and the valve remains closed, the force exerted to open the valve is calculated by multiplying the actuation pressure by the hole area. Conversely, the force applied to close the valve is equal to the total area of the diaphragm in contact multiplied by control pressure (Fig. 3B). Hence, the valve can exert enough force toward closure, even if the control pressure is lower than the actuation pressure. We introduced a rigid disk at the center of the diaphragm to ensure the amplification effect. A fully soft diaphragm tends to deform into a concave shape (Fig. 3B), reducing the contact area. Adding a rigid disk at the center prevents this undesired deformation. If the disk is too large, then it increases diaphragm stiffness and demands higher pressure for closure. Thus, the disk needs to be just large enough to prevent concave deformation. A 2-mm hole was used for a sufficient flow rate, while remaining small relative to the diaphragm size. Accordingly, on the basis of simulations, we used a 4-mm-diameter disk (see the “Guidelines for choosing valve design parameters” section). The remaining design parameters were selected through a parameter study to ensure reliable valve operation, as summarized in Table 1 (see the “Guidelines for choosing valve design parameters” and “2.5D laser cutting–based layer additive fabrication method” sections and figs. S1 to S5 for a detailed selection process).

**Fig. 3.**
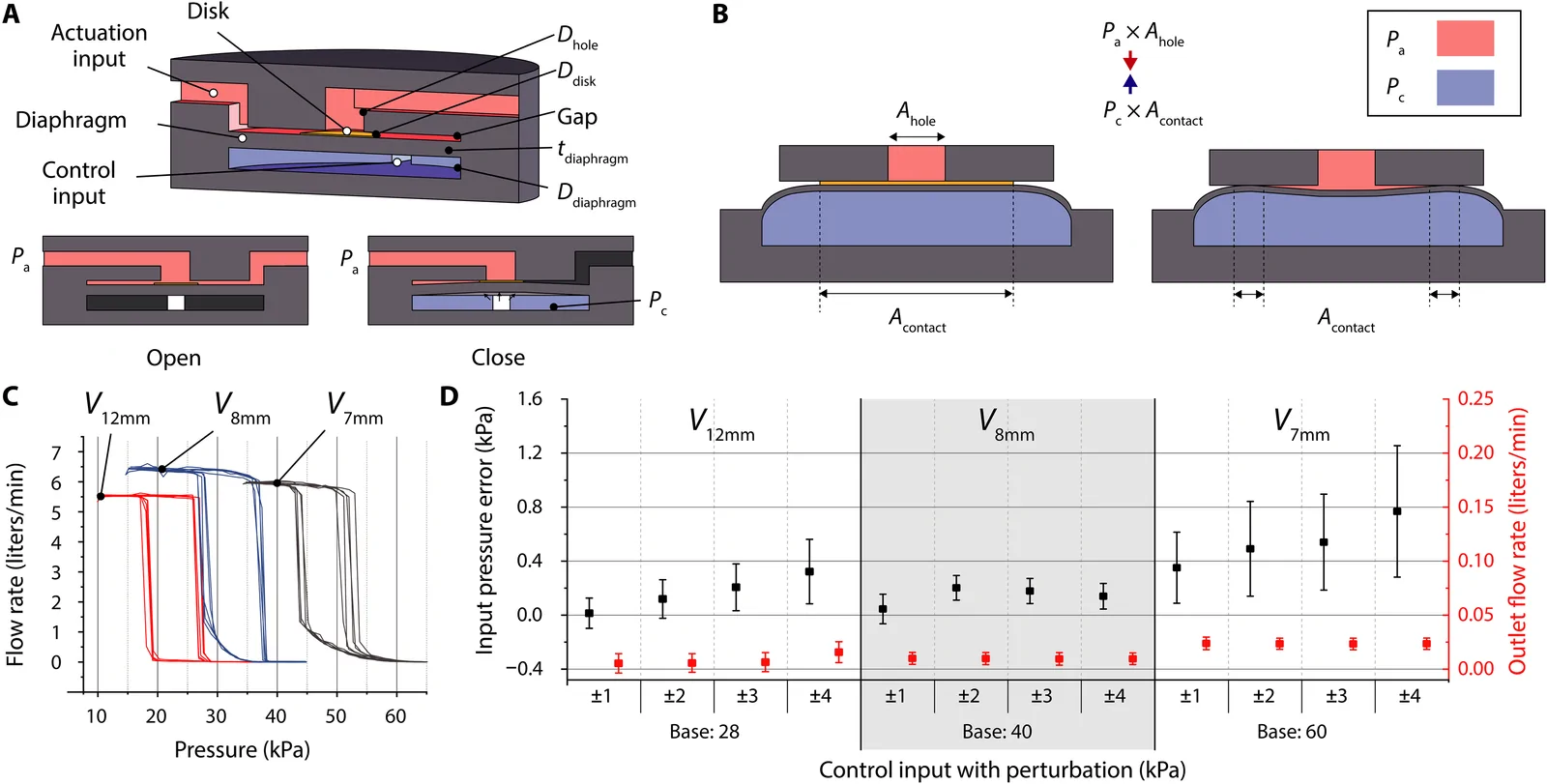
Valve enabling closure under lower control pressure via geometric force amplification. (**A**) The schematic illustration of the valve and its design parameters. (**B**) Mechanical advantage mechanism based on area ratio. The control pressure acts over the full diaphragm area, while the opposing actuation pressure acts over the smaller hole area. This area mismatch amplifies the effective closing force generated by the control pressure, enabling valve closure even when the control pressure is lower than the actuation pressure. The mechanical advantage can be reduced by unwanted concave deformation of the diaphragm. Adding a disk helps prevent this and preserve the amplification effect. (**C**) The transfer function of the selected valves, including five sets of repeated measurements for each valve. (**D**) The flow rate at the valve outlet and the actuation input pressure error when the control pressure experiences a perturbation after the valve is closed. The actuation pressure was set to 50 kPa.

**Table 1.** The selected design parameters.

Design parameters	Value (mm)
Radius of hole ($r_{h}$)	1
Radius of disk (b)	2
Radius of diaphragm (a)	6, 4, 3.5
Thickness of diaphragm (t)	0.75
Height of gap (w)	0.42

Second, repeatability of the valve response was validated by comparing the transfer function of the valve, which describes how control pressure changes affect its behavior (see the “Valve transfer function for valve response analysis” section for the transfer function measurement). The low variation and minimal deviations across trials confirmed the repeatability of the valve response (Fig. 3C). Furthermore, to prevent interference between valve types, the closing pressure of one valve type should not induce unintended partial closure or flow restriction in valves with different closing pressures, which would otherwise manifest as changes in flow rate or increased flow resistance in the measured outputs.

Before discussing interference, it is important to distinguish between the transfer-function flow-shutoff pressure and the minimum operational closing pressure during DEMUX operation. In the transfer-function characterization, the actuation pressure is applied continuously, while the control pressure is swept. Because the outlet is open to atmosphere, flow through the actuation channel causes a pressure drop, so the pressure acting on the diaphragm is lower than the nominal source pressure before sealing. In contrast, the control chamber is closed, so the applied control pressure is transmitted to the diaphragm with negligible pressure loss. Therefore, the valve can shut off flow at a control pressure lower than the nominal source actuation pressure, before mechanical advantage is engaged. This value is defined as the transfer-function flow-shutoff pressure and represents a conservative upper bound for the operational closing pressure. After closing, the boundary condition changes: The upstream actuation region becomes closed volume, and the area difference across the diaphragm generates mechanical advantage that helps maintain closure. As a result, the valve remains closed as the control pressure decreases and reopens at a pressure substantially lower than the closure pressure. This value is defined as the transfer-function flow-release pressure and provides the lower bound for the control pressure required to keep the valve closed after sealing.

During practical DEMUX operation, the control pressure is applied first to close the valve, and the actuation pressure is introduced afterward. Thus, the valve operates with mechanical advantage already engaged. Therefore, the minimum operational closing pressure is better estimated from the transfer-function flow-release pressure, whereas the flow-shutoff pressure serves as a conservative pressure for robust closure. As shown in Fig. 3C, the valve types exhibit separated flow-shutoff and flow-release pressures, preserving distinct operating windows. In valve actuation experiments, conservative command pressures were used to ensure reliable closure, whereas the minimum closing pressure was used for characterization because it more directly reflects the pressure required to maintain sealing during DEMUX operation.

In addition, flow resistance was measured across all control inputs; uniform resistance suggests that geometric variations are minimal and do not affect flow characteristics (see the “Evaluating air delivery capability via relative flow resistance” section and fig. S6). In conclusion, independent operation was confirmed, as closing a valve with low closing pressure did not affect the flow resistance of a valve with high closing pressure (Table 2). We also evaluated the system’s robustness to noise during state transitions by introducing ±1- to ±4-kPa pressure fluctuations at 1 Hz to baseline control pressure corresponding to the closing pressure of each valve. Specifically, the baseline values were 28 kPa for $V_{12\text{mm}}$, 40 kPa for $V_{8\text{mm}}$, and 60 kPa for $V_{7\text{mm}}$. The actuation pressure was maintained at 50 kPa throughout the experiment. Despite slight actuation pressure fluctuations from diaphragm vibrations, the flow rate remained zero, demonstrating reliable closure under perturbations (Fig. 3D). As long as the flow rate stays zero, fluctuations in actuation pressure do not affect the valve’s closed-state flow-blocking behavior.

**Table 2.** The relative flow resistance of the selected valves according to the closing pressure of each valve.

$P_{a}$ = 50 kPa			
$P_{c}$	Relative flow resistance		
	$V_{12\text{mm}}$	$V_{8\text{mm}}$	$V_{7\text{mm}}$
0 kPa	2	1.39	1.60
$P_{\text{closing},V_{12\text{mm}}}$ (28 kPa)	Closed	1.45	1.60
$P_{\text{closing},V_{8\text{mm}}}$ (40 kPa)	Closed	Closed	1.60
$P_{\text{closing},V_{7\text{mm}}}$ (60 kPa)	Closed	Closed	Closed

To implement time multiplexing, we used a bistable structure at each DEMUX output (Fig. 2B and see the “Bistable structure for time multiplexing” section for details on the bistable structure). Positive pressure activates the bistable structure, and it was refreshed using a vacuum.

### Geometric scalability through a model-based scaling design methodology

Changes in size are expected to have limited influence on the valve’s opening and closing pressure thresholds. Preserving them across different sizes is important for maintaining consistent addressing behavior after geometric scaling. Therefore, a design method is required that preserves these opening and closing pressures while enabling geometric scalability. These pressure thresholds are predominantly determined by the position and shape of the diaphragm. When closing the valve, pressure is solely exerted on the control channel, allowing approximation with a model where uniform pressure affects one side of the diaphragm (Fig. 4A). The pressure, q, required to achieve a given vertical deformation w can be expressed in normalized, dimensionless form using a characteristic pressure scale determined by the diaphragm geometry and material properties (see note S4 for the detailed analytical model)

$$q\frac{a^{4}}{w^{3}\mathrm{tE}}=f\left(\nu ,\frac{b}{a}\right)$$ (2)

where t is the thickness of diaphragm, E is the Young’s modulus, ν is the Poisson’s ratio, a is the radius of diaphragm, and b is the radius of disk. Therefore, by maintaining these dimensionless parameters, the valve exhibits the same pressure deformation behavior independent of the absolute device dimensions. In this work, maintaining identical operating pressures across different scales is achieved by preserving the following three parameters: $a^{4}/w^{3}\mathrm{tE}$, ν,and b/a.

**Fig. 4.**
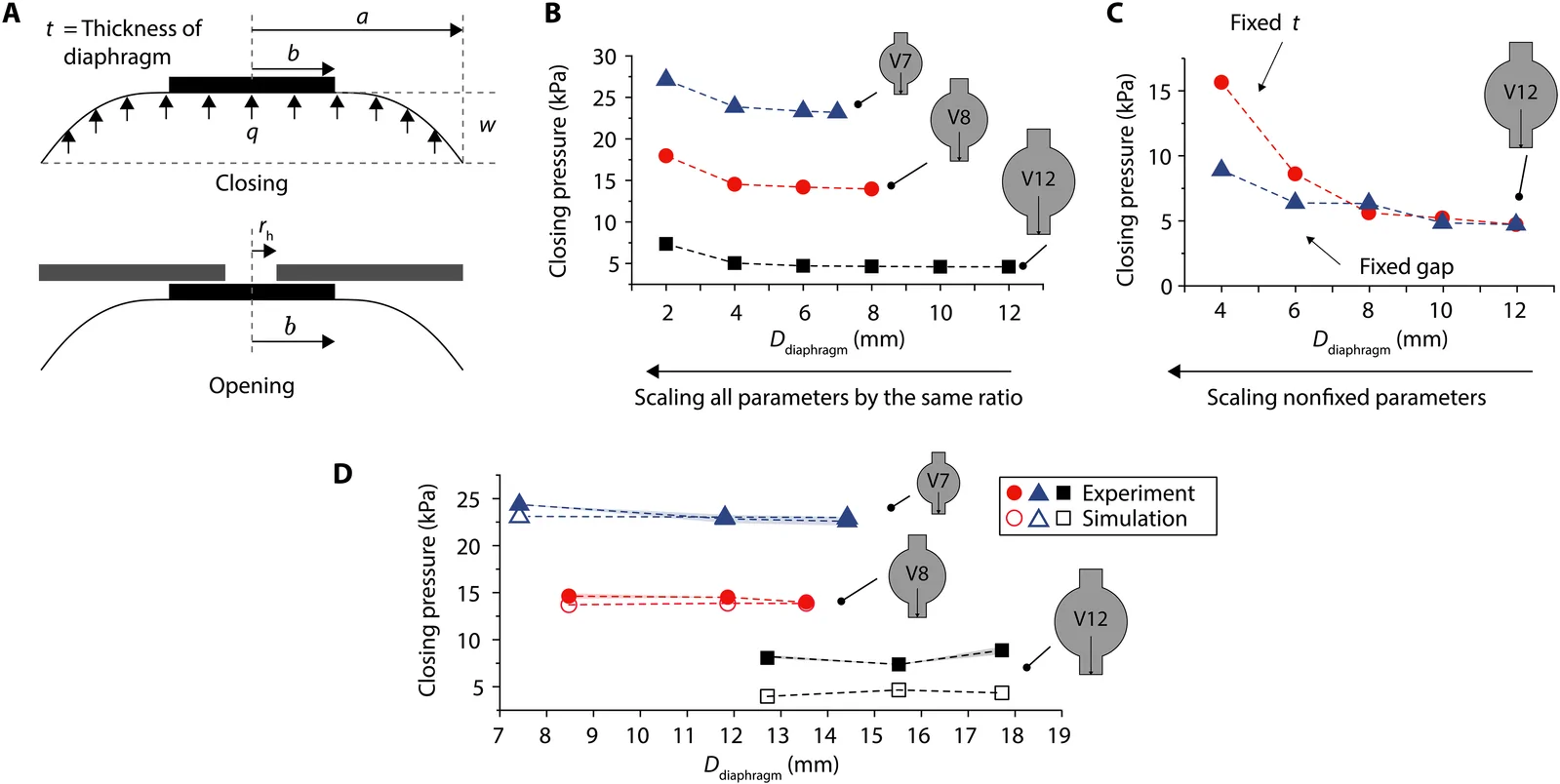
The geometric scalability of the fluidic DEMUX/MUX controller. (**A**) Schematic of the annular diaphragm with a rigid disk at the center with design parameters influencing diaphragm deformation under pressure for closing and opening each. (**B** and **C**) The simulation results showing the closing pressure of the valves at different scales, scaled according to design rules to maintain derived dimensionless parameters. (B) Simulation results for the case in which all geometric parameters of the selected valves are scaled down proportionally. (C) Simulation results for a valve with a 12-mm diaphragm diameter, showing cases where either the gap is fixed at 0.25 mm or the diaphragm thickness is fixed at 0.75 mm, while other parameters are scaled to preserve the dimensionless design criteria. (**D**) Experimental and simulated closing pressures of geometrically scaled valve designs. Filled markers indicate experimental results, hollow markers indicate simulation results, and dashed lines are included only as visual guides.

The geometric scalability derived from the dimensionless formulation is obtained under several simplifying assumptions: linear elastic material behavior, negligible thickness variation during deformation, small–rotation angle assumption (sinθ≈tanθ), and ideal boundary conditions including perfect outer-edge clamping and a rigid central plate without attachment compliance. These assumptions were used to keep the analytical model simple and to provide a practical scaling guideline, rather than to predict the exact closing pressure of each valve design. The model therefore identifies how geometric parameters should be adjusted during scaling to maintain comparable closing and opening pressure ranges. This approximation is suitable because the valves operate as discrete on/off elements, so the closing pressure only needs to remain within a usable operating window.

Once the valve is closed, air enters the actuation line, and the opening is driven by the force difference, determined by the pressure-applied areas (Fig. 4A). To preserve the amplification effect, the area ratio should remain constant.

$$\frac{A_{\text{opening}}}{A_{\text{closing}}}=\frac{\pi {r_{h}}^{2}}{\pi b^{2}}$$ (3)

We first validated through simulations that maintaining the derived dimensionless parameters preserves closing pressure (see the “Simulation conditions” section for simulation details). First, we uniformly scaled down all parameters (see table S4 for detailed size of valve). As a result, we observed consistent closing pressures maintained across different valve sizes (Fig. 4B). Next, we fixed individual parameters while adjusting the others to preserve the dimensionless parameters (Fig. 4C and see table S4 for detailed size of valve). When the gap was held constant, the closing pressure was preserved across scales, whereas fixing the diaphragm thickness resulted in increased closing pressure as the valve was scaled down. The increase in closing pressure during downscaling was attributed to the reduced diaphragm diameter, which made the thin-membrane assumption less valid when the diaphragm thickness was fixed. These results indicate that preserving this assumption is important for maintaining consistent valve behavior across scales. Therefore, diaphragm thickness should be selected carefully in scaled valve designs to ensure that the thin-membrane assumption remains valid.

To compare the simulation results with experimental data for geometric scaling, we fabricated three larger-scale valves for each valve type based on the dimensionless design model (Fig. 4D). The valve dimensions were scaled by applying the same scaling ratio to all geometric parameters. However, because the achievable gap and diaphragm thickness were constrained by our in-house fabrication process, these parameters were slightly adjusted to the closest manufacturable values. The remaining geometric parameters were then recalculated to preserve the derived dimensionless relationships (see table S4 for detailed size of valve). To reduce fabrication-induced dimensional errors during the experiment, we replaced the selected noncontact-critical components with three-dimensional (3D) printed parts. The diaphragm and diaphragm-contacting surface were kept as thermoplastic elastomer (TPE) components to preserve the contact characteristic. This modification enabled a more direct comparison between the analytical model, simulation, and experimental results. As shown in Fig. 4D, the experimental closing pressures closely matched the simulation results, and the scaled valves maintained comparable closing pressures across different sizes. These results support the validity of the dimensionless scaling approach for preserving valve closing behavior after geometric scaling. In addition, we assessed fabrication reproducibility by selecting one valve design, $V_{8\text{mm}}$ size 2, and fabricating three nominally identical samples. The measured closing pressures were consistent across samples, with an overall value of 15.82 ± 0.85 kPa, indicating reproducible valve performance across repeated fabrication (table S5).

While the closing pressure of individual valves is geometrically invariant, the dynamic response of the full DEMUX/MUX system is governed by pneumatic transmission effects. The signal propagation delay can be approximated by a pneumatic time constant τ=RC, where R represents the channel flow resistance and C represents the effective compliance arising from chamber volume, air compressibility, and membrane elasticity. Under geometric scaling of all dimensions by a factor η, channel resistance scales approximately as $R∼\eta^{-3}$, as described by the Hagen-Poiseuille relation, while compliance scales as $C∼\eta^{3}$, due to volumetric scaling, leading to an approximately scale-independent characteristic time constant.

### Physical integrability through structural compliance matching

Stable flow resistance, defined as the ratio of pressure drop to volumetric flow rate, is crucial for integrating fluidic circuits into a deformable body. In addition, the circuit’s deformability is key to preserving actuation behavior after integration. To achieve this, we connected each valve to structurally stretchable channels made from relatively stiff materials, with a Young’s modulus approximately 5 to 10 times higher than that of the base soft actuator material, which prevents bulging and resists external forces such as bending and compression. The channels accommodate stretching through rotation and translation, with tortuosity greater than one (Fig. 5A). A horseshoe shape was chosen for its uniform stress distribution and robust behavior under strain, allowing the fluid to follow a smoothly curved flow path without inducing kinking or flow obstruction (*39*–*42*). The channel width in a horseshoe structure is a critical design parameter, directly influencing flow resistance and pressure-holding characteristics. It depends on both the fluidic cross section and the bonding width, with a minimum bonding width of 1.5 mm required for reliable layer adhesion (see the “2.5D laser cutting–based layer additive fabrication method” section and note S5). The fluidic cross-sectional area determines the actuation speed across the system. This speed is particularly important in DEMUX design, as it influences the timing delays between the closest and farthest channels (Fig. 5B). We analyzed the relationship between cross-sectional area and actuation speed by measuring the relative flow resistance of each valve and channel and calculating the maximum number of addressable outputs within a specified delay limit (see the “Evaluating air delivery capability via relative flow resistance” section). With a channel width and height of 1 mm and the actuation input positioned at the center of the circuit, the current design can effectively operate a four-quaternary input DEMUX with a 350-ms delay between the nearest and furthest outputs. The acceptable delay varies by application, and channel width is being selected according to the desired actuation speed.

**Fig. 5.**
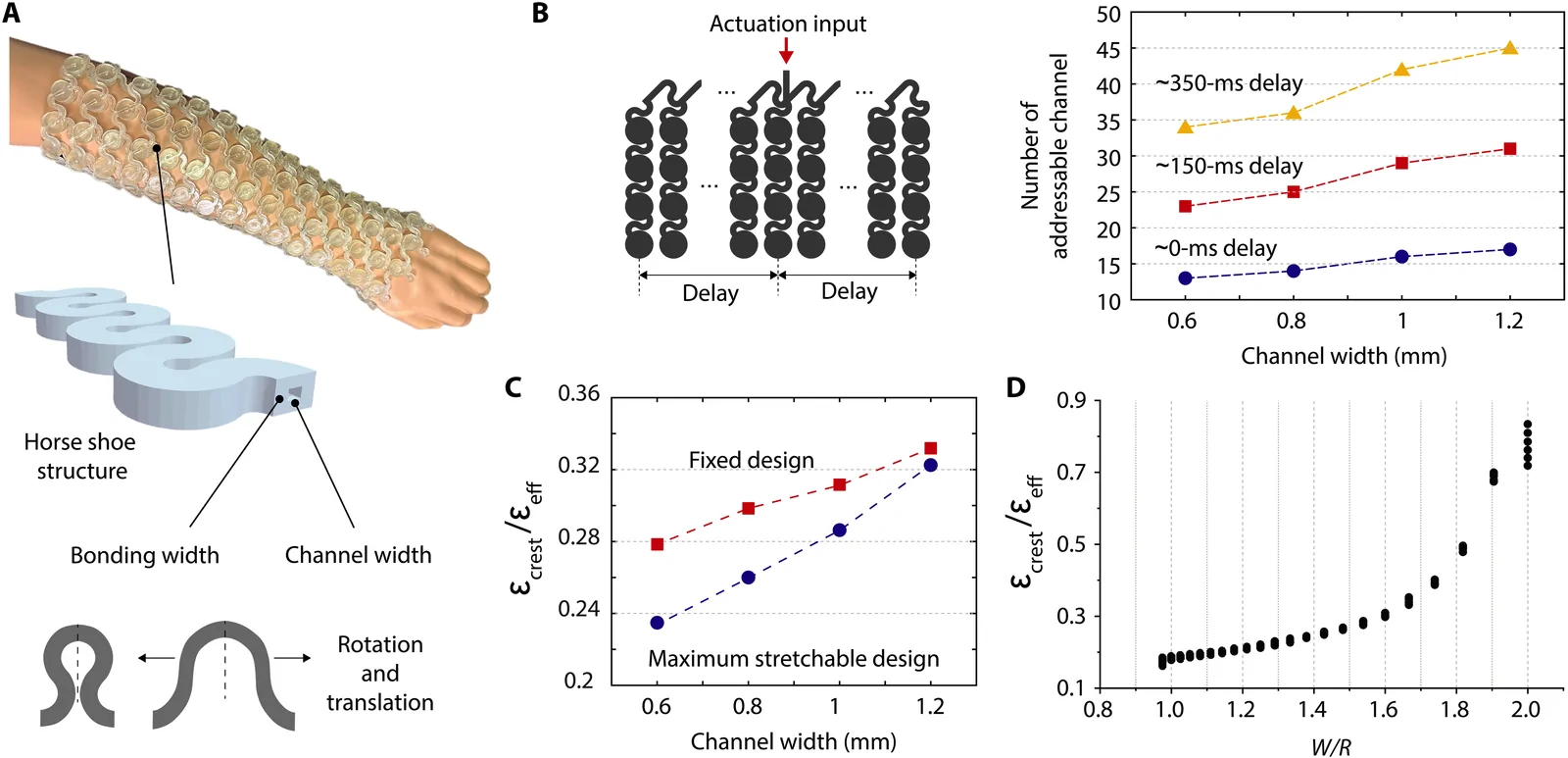
Physically integrable pneumatic channel with structural compliance. (**A**) Schematic illustration of horseshoe-shaped channel. (**B**) The maximum number of addressable outputs within a specified allowable delay for different channel widths. (**C**) The stretchability of channel according to the channel width. The blue data series represents the maximum stretchability achievable with the given channel width. The red data series shows the scenario where we kept the design parameter constant regardless of changes in width (detailed design parameters are shown in note S6). (**D**) A comparison of stretchability in designs that meet mechanical constraints. The variation among designs with the same W/R ratio is due to different L/R ratios.

Delay is critical for controller timing because it can constrain the achievable controller bandwidth. However, from the soft robot’s standpoint, stretchability is equally important to minimizing the impact of controller integration on actuator motion. Here, we use the dimensionless ratio $\epsilon_{\text{crest}}/\epsilon_{\text{eff}}$, where $\epsilon_{\text{crest}}$ is the maximum strain at the crest of the horseshoe structure and $\epsilon_{\text{eff}}$ is the effective applied strain of the overall structure, to compare stretchability, as the crest part experiences the maximum strain. A lower value indicates greater stretchability, while a higher value suggests less stretchability. It is evident that with a fixed serpentine design, increasing the width leads to decreased stretchability. However, while we adjust the serpentine design based on channel width to maximize stretchability under given conditions, a larger cross-sectional area reduces stretchability (Fig. 5C). Therefore, a balance must be struck to optimize both factors.

Given that most fluidic soft actuators operate at a typical driving frequency of 1 Hz (*17*), we selected a 1 mm–by–1 mm channel cross section for this research, with a total width of 4 mm. Following design guidelines for stretchable electrical interconnects (see note S6 and fig. S7), we examined the conditions with the greatest impact when the width is fixed. Using a 4-mm channel width, we identified geometries that satisfied both the nonoverlap and vertical size constraints and then simulated each case at 100% strain to calculate $\epsilon_{\text{crest}}/\epsilon_{\text{eff}}$. Results showed that combinations with a smaller W/R ratio greatly enhanced stretchability, where W is the channel width and R is the curve radius (Fig. 5D). As the W/R ratio increased, so did the L/R ratio, where L is the arm length, resulting in higher strain at the crest. Therefore, selecting combinations with smaller W/R ratio leads to a more stretchable structure. On the basis of these findings, assuming a channel width of 4 mm and a valve diameter of 18 mm, we identified the parameter combination that provides the highest stretchability (Table 3).

**Table 3.** Design parameters of selected curved channel.

Design parameter	Width of channel, *W* (mm)	Length of arm, *L* (mm)	Radius of curve, *R* (mm)	Angle of curve, θ (°)
Value	4	2	4	119

### Flow resistance stability of distributed circuits on soft body under external loading

We demonstrated the ability of the stretchable interconnects to maintain stable flow resistance when attached to a soft body and subjected to external loading. First, we compared the changes in flow resistance under stretching and bending. For this comparison, we fabricated a linear channel with the same projected length and cross-sectional area using a material seven times softer than TPE (Dragon Skin 10, Smooth-On Inc.). Both channels were connected to a linear guide, widening them to induce stretching and narrowing them to induce bending (Fig. 6A). The experimental range for stretching was limited to 50-mm displacement, as finite element simulations indicate that larger deformations lead to considerable geometric distortion and reduction of cross-sectional area. Beyond this range, the cross-sectional area decreases to ∼90% or less of its initial value, resulting in nonlinear increases in flow resistance. Although the linear channel showed lower flow resistance in its undeformed state, resistance substantially rose when subjected to deformation (Fig. 6B and see the “Evaluating air delivery capability via relative flow resistance” section). Conversely, the curved channel consistently maintained stable flow resistance even under deformation, indicating reduced sensitivity to stretching and bending.

**Fig. 6.**
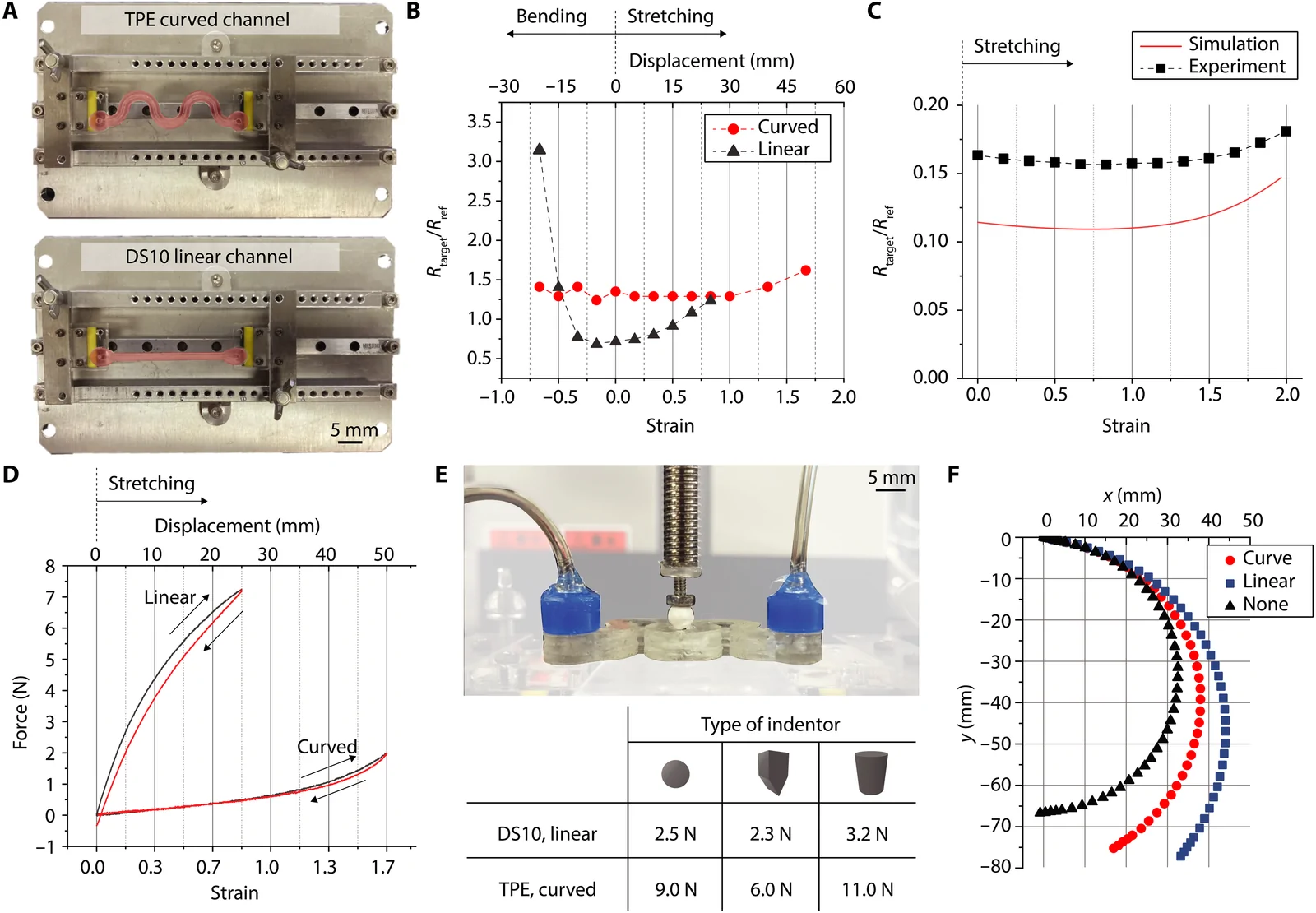
Stable flow resistance of deformable channels under different loading: stretching, compression, and bending. (**A**) Two samples: a curved TPE channel and a linear Dragon Skin 10 channel. (**B**) Displacement–flow resistance curve of the two samples. Positive strain indicates stretching, and negative strain indicates bending. (**C**) Comparison of simulated and experimental flow resistance of the curved channel under stretching. Markers indicate discrete tested conditions; dashed lines are included only as visual guides. (**D**) Displacement-force curve of the two samples. (**E**) Force required to achieve the same flow resistance level tested with different indenter shapes. (**F**) Finite element simulation results comparing the bending curvature of the soft robot under the same pressure input, without channel integration and with integration of the two different channel designs.

To further evaluate the stretch limit of the curved channel, we compared the experimentally measured flow resistance with the resistance estimated from the finite element analysis (FEA) (Fig. 6C). In this test, the curved channel was stretched up to a strain of 2. The simulation and experiment show the same overall tendency: The flow resistance remains relatively stable at small to moderate stretch but increases more clearly at larger deformation. The sharp increase in flow resistance at larger stretch can be explained by a transition in the dominant deformation mechanism. At small stretch, the curved channel mainly rotates and straightens, so the internal cross section changes only slightly and the resistance remains nearly constant. At larger stretch, after the curved geometry has opened, axial stretching of the elastomeric wall becomes dominant. This causes transverse contraction, reduces the internal channel opening, and leads to a sharper increase in flow resistance. This result indicates that, to maintain an approximately constant flow resistance during stretching, the practical operating strain should be kept around or below 1.5.

Although the simulation captures the overall trend, it underestimates the absolute resistance because it calculates only the resistance of an idealized channel geometry. In the experiment, the measured resistance also includes unavoidable losses from connectors, tubing connections, and inlet/outlet regions. In addition, small fabrication–induced variations, such as local narrowing, wall deformation, bonding deformation, or cross-sectional asymmetry, can increase the resistance. This effect is amplified because hydraulic resistance is highly sensitive to channel height and scales approximately with $h^{-3}$. Therefore, even small reductions in local channel height can produce a large increase in the measured resistance.

In addition, we examined how the channels respond to external forces. The horseshoe structure reduces channel stiffness while limiting material strain, which contributes to the decreased hysteresis observed in Fig. 6D. To evaluate the resilience of channel to external forces, we performed comparative analyses on the force necessary to reach equivalent resistance levels (see the “Experiment comparing channel resistance to external force” section). We used indenters for applying load in varying shapes (sphere, wedge, and cylinder) to exert force exclusively on the channels (Fig. 6E). Comparisons were made between the curved and linear channels, the same ones used in the previous experiment. The curved design showed higher force resistance, with the high-modulus sample demonstrating over twice the force resistance compared to the low-modulus channel.

When integrating the circuit into a soft robot, it should not only be robust to external loading but also minimize its impact on the robot’s movement. Therefore, we conducted simulations to investigate the effects of integrating channels into a bending soft robot (see fig. S8 for detailed information about the bending soft robot). Our findings revealed that the curved design had notably less impact compared to the linear channel, although it has a higher material modulus (Fig. 6F). In addition, we investigated the effect of actuator deformation on the channel. Initially, the cross-sectional area of the channel was 1.2 mm^2^. Under similar bending angles of the actuator, we observed minimal changes in the curved structure (1.24 mm^2^), while the linear channel experienced a substantial 40% reduction in area (0.75 mm^2^) (see fig. S8). These results highlight the advantages of the curved channel, as it had a lesser impact on the motion and affected the actuation speed to a lesser extent, an important factor for controller timing.

### System-level demonstration of scalable and integrable fluidic DEMUX/MUX

We demonstrated the fluidic DEMUX/MUX controller concept by building a four-quaternary input DEMUX with a bistable structure at each DEMUX output (Fig. 2, A and B). This DEMUX controls 40 individual outputs, divided into four sections of 10 outputs each for practical convenience. Each section measures 250 mm by 105 mm by 7 mm and weighs ∼80 g (Fig. 7A). For demonstration, we controlled a digital panel display to render a desired shape. A section with 10 outputs was selected and connected to a 4 pixel–by–3 pixel array, including two nonfunctioning pixels and 10 active pixels (Fig. 7A). Each bistable structure was located to push the panel, tilting it to create an angled position. The display represents binary states: “on” when the panel is angled and “off” when it remains flat. The activated bistable structures can be reset using a vacuum signal applied to the actuation line. By connecting bistable elements located at each DEMUX output to different types of digital-state actuators, various shape transformations can be achieved. All demonstrations described in this section are shown in movie S1.

**Fig. 7.**
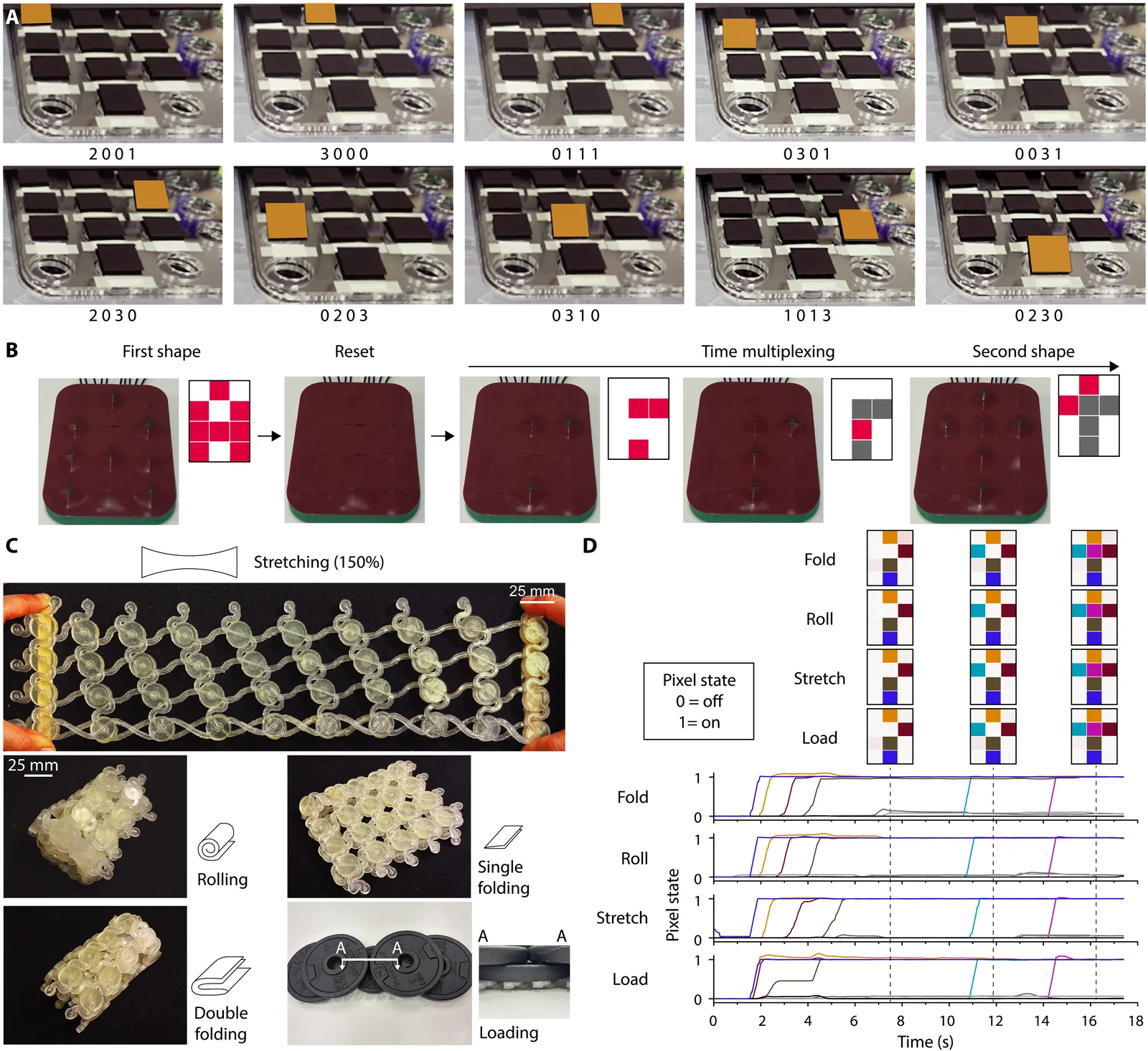
The demonstrations with fluidic DEMUX/MUX using four quaternary inputs. (**A**) The selection of a single channel. (**B**) Demonstration of reset and rewriting together with time multiplexing. (**C**) The various deformation states. (**D**) Time traces of pixel activation states during time-multiplexed operation under different deformation conditions. The *y* axis represents the binary activation state of each pixel (0 = off and 1 = on), and each colored trace corresponds to a different pixel in the pixel map shown above the graph. The same color coding is used for the pixel map and the corresponding time traces.

#### Validating scalability through single-channel selection and shape rendering

We first demonstrated the system’s ability to individually select each channel. As shown in Fig. 7A and movie S1, each pixel can be activated independently, confirming that any pixel combination is achievable when time multiplexing is combined. Before pattern generation, all outputs are simultaneously reset to the inactive state using a vacuum signal, while all valves are open, thereby establishing a well-defined initial condition.

The selected section with 10 outputs can generate 38 unique primary patterns, as determined using the method described in note S2. Figure 7B illustrates representative shape-rendering examples. An initial “A” shape is generated and then fully reset using a vacuum signal, followed by rewriting an upward-pointing arrow through the combination of multiple primary patterns. The arrow configuration, requiring activation of six pixels, was achieved using three sequential input sets. Each frame in Fig. 7B corresponds to the system state after completion of one input session. To determine the minimum set of primary patterns required to generate a desired output, we apply an iterative selection procedure. First, we filter the available primary patterns to retain only those whose inactive regions coincide with the inactive regions of the target pattern, thereby ensuring that no unintended pixels are activated. Among the filtered candidates, we select the pattern that activates the largest number of pixels required by the target. The pixels activated by this selected pattern are then removed from the target configuration, and the updated configuration is treated as the updated target. This procedure is repeated until all desired pixels are activated and the complete pattern is obtained. The algorithm is described in detail in note S3. The final result was achieved by sequentially introducing and activating the chambers based on the derived inputs.

Under the defined reset condition, the mapping from an input sequence to the final pixel state is deterministic. However, because the outputs are bistable and retain their state after activation, multiple input sequences may produce the same final pixel configuration. Therefore, the reverse mapping from a given pixel state to an input history is not necessarily one to one; instead, the addressing algorithm provides one minimal input sequence capable of generating the desired pattern.

#### Physical integrability validation through actuation under loading

We demonstrate that our DEMUX/MUX controller maintains stable output generation under external loading while preserving the key advantage of soft robots: adaptability to unconventional shapes. These deformations are made possible by the stretchable interconnects, which provide the circuit with flexibility and softness, enabling it to stretch, roll, and fold. As illustrated in Fig. 7C, the circuit can be stretched up to 150% and can easily accommodate rolling and folding. To test its robustness under external loading, we repeated the shape-rendering process while exposing the circuit to folding, rolling, stretching, and loading (Fig. 7, C and D). The results, shown in Fig. 7D, demonstrate that the system performed as expected, producing the desired outputs using the same input combinations, even with these four external loadings. Therefore, we conclude that when the circuit is integrated into a soft robot body, the typical disturbances encountered by soft robots are unlikely to compromise its actuation functionality.

## DISCUSSION

This study presents a scalable and integrable fluidic control architecture that addresses three central challenges in soft robotics controllers: functional scalability, geometric scalability, and physical integrability. Functional scalability was achieved by integrating a high-radix DEMUX with time multiplexing. The use of multistate inputs increased the number of addressable outputs greatly without expanding the input lines, supporting a more compact and efficient design. Specifically, four quaternary inputs controlled up to 40 independent outputs. The bistable mechanical memory elements at each DEMUX output enabled complex actuation states via time-multiplexed activation by retaining each output state after transient addressing. This architecture allows diverse, reprogrammable actuation patterns without the need for extensive external connections, preserving the inherent flexibility of soft robotic systems. To ensure geometric scalability, a design methodology based on dimensionless parameters was introduced. This approach allows the physical dimensions of the valves and circuits to be adjusted while maintaining comparable valve opening and closing pressure thresholds and preserving consistent DEMUX addressing behavior. By maintaining key geometric ratios derived from diaphragm and channel models, we demonstrated consistent closing pressures across different valve sizes, facilitating straightforward adaptation of the system to robots of varying scales.

Physical integrability was achieved through the development of structurally stretchable interconnects using stiffer yet flexible materials. These interconnects preserve fluid transport reliability under stretching, bending, and compressive loads, while minimally interfering with the robot’s deformation and movement. The horseshoe-shaped channel design effectively distributed mechanical strain and prevented flow obstruction during deformation, supporting seamless embedding of the control circuits within soft robotic structures.

The introduction of time multiplexing in the proposed system not only substantially expands its functional space but also introduces trade-offs that merit discussion. Even without time multiplexing, the DEMUX/MUX can generate a large number of static output configurations; for example, in the demonstrated device with 40 outputs, 147 unique output patterns can be directly addressed using a single input configuration, which may be sufficient for many applications. Accordingly, in the example shown in Fig. 7, connecting the outputs to different chambers would allow the pattern to be achieved using only a single input setting. Time multiplexing, as demonstrated in Fig. 7, is therefore not used to compensate for an inability to generate specific patterns but rather to further expand the range of achievable configurations by encoding patterns sequentially over time. This capability enables sequential recording, erasing, and rewriting of output states while maintaining a limited number of input channels. However, time multiplexing introduces temporal constraints due to sequential state programming, and, as with most fluidic control architectures, the proposed approach is not intended for high-bandwidth control. These limitations reflect inherent trade-offs between response time, input channel count, and pattern diversity and are common to multiplexed fluidic DEMUX/MUX systems. Overall, this work broadens the functional capabilities of soft robots by enabling multifunctional designs with embedded, scalable control architectures while preserving their inherent compliance and adaptability.

## MATERIALS AND METHODS

### Guidelines for choosing valve design parameters

This section provides a detailed analysis of the key design parameters for a valve, focusing on diaphragm thickness, disk size, and gap. By exploring their impact on deformation, closing pressure, and flow rate, we aim to offer a comprehensive guide for achieving a balanced valve design.

Before selecting the other parameters, we set the diaphragm thickness to 0.75 mm to ensure robustness against fabrication errors while maintaining a target working pressure of around 50 kPa. We first analyzed the case without a disk to approximate the diaphragm’s deformation under pressure. When the circular diaphragm’s edge is fixed and subjected to uniform pressure (fig. S1A), the deformation along the z axis can be expressed as follows

$$\Delta z\left(r\right)=\frac{3p\left(1-\nu^{2}\right)}{16Et^{3}}{\left(a^{2}-r^{2}\right)}^{2}$$ (4)

$$\Delta z\left(0\right)=\frac{3p\left(1-\nu^{2}\right)}{16Et^{3}}a^{4}$$ (5)

Thickening the diaphragm increases the required closing pressure, while widening the gap has a similar effect. However, the diaphragm deformation scales approximately with 1t3, small thickness errors can strongly affect the closing pressure. Thus, we prioritized increasing the diaphragm thickness. However, excessive thickness reduces deformation within the working pressure range, hindering distinct opening and closing actions. To balance precision and functionality, we chose a 0.75-mm-thick TPE diaphragm. This thickness ensures robustness against production errors and provides sufficient vertical deformation even with a small diameter (r = 3 mm), making it suitable for our application (fig. S1B).

Next, the disk size was determined. As discussed in the “Architectures and characterization of hybrid fluidic DEMUX/MUX” section, the disk is used to prevent concave deformation of the diaphragm, which reduces the amplification effect. Therefore, since the amplification effect only takes place after contact is made, it is essential to compare the pressure at which contact occurs with and without the disk. The design aims for full contact, where the edge of the hole touches the disk, and this happens at equal or lower pressure with the disk in place. We conducted simulations to compare the valve’s vertical deformation with and without disks of various sizes at the edge of the hole ($r=r_{\text{hole}}$; fig. S1C). The simulation settings are described in the “Simulation conditions” section, and we evaluated different diameters of diaphragm (D = 7, 8, and 12 mm) and disk sizes ($D_{\text{disk}}$ = 3, 4, 5, and 6 mm). A larger deformation with a disk ensures earlier contact and a higher amplification ratio (fig. S1D). We plotted deformation differences between cases with and without a disk and compared them (fig. S1, E to G). Simulations revealed that a small disk notably increased vertical displacement compared to the disk-free case and effectively prevented concave deformation. Taking into account the predetermined 2-mm hole size and potential fabrication errors, a 4-mm disk was selected for this study.

The remaining variables are the gap and diameter of diaphragm. Diameter, variable on the horizontal plane, is advantageous as a design variable to achieve different closing pressures between valves. In contrast, varying the gaps between valves complicates the manufacturing process and reduces system durability due to the need for additional thin layers. Therefore, we decided to fix the gap at a single value and vary the diameter to differentiate the closing pressure. We created samples with three different diameters (8, 10, and 12 mm) and three gap sizes (0.42, 0.67, and 0.92 mm) and experimentally obtained their transfer functions (see the “Valve transfer function for valve response analysis” section for transfer function measurement process). On the basis of previous results, the disk size was set to 4 mm, and the diaphragm thickness was kept constant at 0.75 mm. A smaller gap not only enables faster contact and lowers the closing pressure (fig. S2, A and B) but also raises concerns about a potential decrease in flow rate. However, since the diameter was sufficiently large and the flow resistance of other parts was greater, the flow rate difference caused by the gap difference was minimal. Moreover, the amplification effect becomes notable from the moment of contact, making a smaller gap even more advantageous. Therefore, a gap of 0.42 mm was chosen for this study, focusing on observing distinctive closing pressures and low hysteresis in valves with diameters of 12 and 8 mm.

For the maximum case of quaternary input, three types of valves with different closing pressures are required. The analytical model (Eq. 5) shows a power law dependence of closing pressure on diaphragm radius, with smaller diaphragms producing substantially higher closing pressures. This diameter effect is much stronger than the effect of increasing gap height, indicating that diaphragm diameter is the primary geometric parameter governing valve closing pressure (fig. S2B). On this basis, we added a valve with a diameter of 7 mm to the previously tested 8 and 12 mm to achieve adequate closing pressure differences between the valves (fig. S2C).

### 2.5D laser cutting–based layer additive fabrication method

The 2.5D laser-cut layer-by-layer fabrication method (fig. S3A) was used to achieve geometric scalability by precisely controlling the controller’s size and complexity, which stems from its large number of valves. Adapted from a prior fabric-based approach (*43*), this method leverages laser cutting for high-precision control in the xy plane, a large working area, and material versatility (*44*, *45*). Unlike conventional methods, it integrates cutting and layer alignment simultaneously, eliminating manual postprocessing and improving accuracy, especially important for soft, complex, multiscale structures. The cut-and-stack process is repeated with the laser tuned to cut only the newly stacked layer (see the “Tuning laser parameters for cutting depth and precision” section). TPE was selected for its ease of bonding via heat pressing (see the “Thermoforming and bonding ability of TPE” section). This method suits the plunge-type valve, which can be separated into planar layers. Given that the DEMUX contains multiple valves, fabrication repeatability is critical for maintaining consistent valve switching behavior. We compared the closing pressures of three separately fabricated samples. Although there were slight variations in closing pressure, the differences between valves of various sizes were substantial enough to avoid any interference (fig. S3B).

#### Tuning laser parameters for cutting depth and precision

This section details the process of laser parameter tuning, focusing on optimizing cutting depth and precision for TPE material. Accurate depth control in laser cutting requires determining parameter values that achieve precise penetration, which vary on the basis of material properties and laser cutter specifications. We used a 60-W laser cutter (Speedy 300, Trotec) with a maximum cutting speed of 355 cm/s. For TPE, the material used in this study, vector cutting was applied with a fixed speed of 3.5 cm/s and a laser frequency of 2000 Hz, while the cutting depth was adjusted by varying the number of paths and laser intensity. To determine the optimal parameters, we measured penetration depth and cutting width under varying path counts and power levels (see table S6 and fig. S4A). Penetration depth is critical for ensuring the cut matches the target layer thickness, while cutting width affects fabrication precision, making a narrower width preferable when depth remains unchanged. Increasing the number of paths reduced the laser power needed for the same depth, but the effect was most pronounced when increasing from one to two paths (see fig. S4B). Cutting width showed a slight but consistent decrease (see fig. S4, A and B). Although using multiple weak-intensity paths improved precision, it substantially increased cutting time. Since this study aimed to produce large-area samples, time efficiency was prioritized. The reduction in cutting width (∼0.1 mm) was not sufficient to justify the 2 to 3× increase in processing time, so a single-path approach was chosen. For single-path cutting, a linear fit of cutting depth to laser power allows for estimating the required power for future cuts. While fine-tuning is necessary for initial setups, this method provides a reliable starting point for setting power levels.

Required laser power = 65 × Desired cutting depth+4 (6)

To verify the equation, a comparison was made between the cutting depth obtained from the formula-derived laser power and the desired cutting depth by actually performing cuts. Although some errors were observed, as mentioned earlier, the equation demonstrated sufficient accuracy for gross tuning (see table S7 and fig. S4C).

#### Thermoforming and bonding ability of TPE

This section discusses the use of TPE in constructing the circuits, highlighting its excellent bonding and thermoforming properties, which simplify the bonding process and enable efficient rapid prototyping. Specifically, in this research, we used a block copolymer of TPE (Flexdym, Eden Tech) and polydimethylsiloxane, a biphasic material combining the advantages of thermoplastic and elastomer properties. To achieve the desired thickness, we heat pressed the material at 180°C for 2 min at 10 bar, following a thickness guide. While conformal contact at room temperature is sufficient for bonding, we expedited the process using heat pressing at 85°C for 2 hours at 10 bar. At this temperature, the polymer maintains a rigid block state, preventing deformation (*46*). To ensure uniform bonding, a guide was used to maintain distance between the two heat plates. Through bonding pressure measurement tests, we obtained bonding pressures exceeding 140 kPa when the bonding width exceeded 1.2 mm (see note S5 and fig. S5). Considering that the typical input pressure for soft robots is ∼103.4 kPa (*17*), our bonding strength surpasses the soft robot’s input pressure range. In this study, with fabrication completeness in mind, we aim to set the wall thickness to values exceeding 1.2 mm.

### Valve transfer function for valve response analysis

This section describes the process of deriving the valve’s transfer function, which defines the relationship between the control pressure and the resulting output flow rate.

To deduce the transfer function of the valve, we measure the output flow rate according to the control pressure. The output of a valve changes with the control input, and while an ideal situation results in an inverted step function, there is often a transition region. A steeper transition region allows for more precise closing, making it ideal. The input pressure refers to the pressure of the control line that determines the movement of the diaphragm, and the output can be either the input pressure of the actuation line or the output flow rate. When the valve opens, the input pressure is measured to be lower than the source pressure due to the pressure gradient between the source and end point. However, a closed volume is formed when the valve is closed, and the pressure measured is the same as the source pressure. As for the output flow rate, when the valve is open, air flows continuously, and the flow rate is measured. On the other hand, when the valve is closed, the air cannot pass, and the flow rate cannot be measured. Both of these variables are sufficient to define the opening and closing of the valve. In this study, we used the output flow rate as the output variable because our primary objective was to evaluate the valve’s flow control capability, specifically its opening and closing behavior, rather than its pressure regulation performance. Flow rate measurements also allowed us to examine whether the opening or closing of one valve influences the flow characteristics of the other valves, particularly their flow resistance. Because flow resistance is defined as the pressure difference divided by the flow rate, we maintained constant pressure boundary conditions by applying the same source pressure and exposing the outlet to atmospheric pressure. Under these conditions, changes in the measured flow rate indicate changes in flow resistance. The actuation input pressure was fixed at 50 kPa, and the control pressure was increased in increments of 1 kPa using an electropneumatic regulator (ITV-1011, SMC, Japan). The flow release pressure was extracted from the pressure-flow response of each valve actuation cycle using a local piecewise linear intersection method. Pressure and flow rate were recorded simultaneously, while the valve was swept through closed and open states. For each cycle, the release event was first identified as the sharp transition from the low-flow closed state to the high-flow open state. A linear fit was then applied to the two data points spanning this abrupt transition, representing the local release trend. Separately, a second linear fit was applied to the low-flow region immediately before release, representing the closed-valve baseline. The flow release pressure was defined as the pressure coordinate at which these two fitted lines intersected.

### Evaluating air delivery capability via relative flow resistance

This section outlines the method used to evaluate the system’s air delivery capability by measuring relative flow resistances. We measured relative flow resistances rather than absolute values, as mass flow rate offers a more accurate measure of flow resistance in pneumatic systems, but accurately quantifying it at a small scale is challenging (*47*). Thus, we eliminated the mass flow rate term from the equation using relative resistance. Furthermore, by consistently comparing the samples to a fixed reference channel, we can express their flow resistance relative to the reference channel’s specific length. This substitution can simplify the experiment and facilitate the comparison (an applied case of this substitution is presented at the end of the section). The experimental setup is depicted in fig. S6. Initially, we assumed isothermal, laminar, subsonic flow of an ideal gas, for which density is proportional to absolute pressure. All pressure values used in the pressure-squared formulation were converted to absolute pressure. In this case, the flow resistance R is defined as follows

$$R=\frac{P_{\text{inlet}}-P_{\text{outlet}}}{\dot{V}}$$ (7)

$$R\dot{m}={P_{\text{inlet}}}^{2}-{P_{\text{outlet}}}^{2}$$ (8)

where $\dot{V}$ is the volumetric flow rate and $\dot{m}$ is the mass flow rate. When the reference channel and the sample are connected in series, the mass flow rate through both channels becomes the same, and we can cancel out them when we obtain a resistance ratio.

$$\frac{R_{\text{target}}}{R_{\text{ref}}}=\frac{{P_{\text{inlet}}}^{2}-{P_{\text{atm}}}^{2}}{{P_{\text{source}}}^{2}-{P_{\text{inlet}}}^{2}}$$ (9)

In this research, we used a 200-mm length of polyurethane tube with an outer diameter of 2 mm and an inner diameter of 1.2 mm as a reference channel. The source pressure was maintained at $P_{\text{source}}$ = 50 kPa for all experiments, except for those comparing flow resistance in the bent and stretched states, where $P_{\text{source}}$ = 100 kPa was used (Fig. 6B).

#### Using relative flow resistance for comparison

To estimate the delay between channels in the “Physical integrability through structural compliance matching” section, we substituted actual valves and interconnects with commercial tubes of specific lengths that provide equivalent resistance using relative flow resistance. Representing valves and interconnects as corresponding lengths of a reference channel, we treated each valve-interconnect pair as a single unit and compared four different cross sections: squares with side lengths of 0.6, 0.8, 1.0, and 1.2 mm. By using relative flow resistance to estimate the delay between channels, we can replace the actual valves and interconnects with commercial tubes of specific lengths that offer equivalent flow resistance. We represented the valves and interconnects as specific lengths of reference channels, using relative flow resistance.

This substitution allows the effective channel length to be adjusted in a straightforward and systematic manner, enabling investigation of signal delay independently of specific valve geometries or interconnect shapes. Moreover, representing all components using a standardized reference channel makes the results broadly applicable, allowing delay behavior to be predicted for different device configurations and scaling scenarios even when valve dimensions or channel curvature is modified. Treating a single set of unit interconnect and a valve as a unified unit, we compared four different cross-sectional cases: squares with side lengths of 0.6, 0.8, 1.0, and 1.2 mm. The approximated delays observed are as follows: 50 ms for 250 cm of reference channel, 150 ms for 450 cm of reference channel, and 350 ms for 650 cm of reference channel. To determine the number of addressable channels, we reverse engineered the reference channel length to calculate how many valve and interconnect units can fit.

### Bistable structure for time multiplexing

This section describes the bistable structure we used to achieve time multiplexing capability. The bistable structure is illustrated in Fig. 1C, with a diameter of 20 mm and a maximum activation height of 5 mm. We used heat molding to shape the bistable structure. We fixed a 0.75-mm-thick layer to the 20-mm–inner diameter and 30-mm–outer diameter acrylic guard and then applied heat to mold it according to the template. The snap-through pressure from off to on was around 50 kPa.

### Simulation conditions

This section describes the simulation conditions used for FEA in ANSYS, which were used to compare valves with different parameters and gain a better understanding of their deformation behavior and closing-pressure response under varying conditions. In the finite element simulations, the TPE material behavior was modeled using an Ogden hyperelastic model fitted to the experimental stress-strain data. Over the strain range of 0.017 to 0.855, the fitted Ogden model closely matched the experimental response, with a normalized root mean square error of 0.0229. For Dragon Skin 10, Yeoh model with following parameters were used: $C_{1}$ = 36 kPa, $C_{2}$ = 0.25 kPa, and $C_{3}$ = 0.023 kPa.

#### Simulation of diaphragm deformation with and without disk

The pressure inside the chamber was varied, while the pressure applied to the top of the diaphragm was kept constant at 50 kPa, with the affected area limited to the size of the hole (see the “Guidelines for choosing valve design parameters” section).

#### Simulation of scalability of valve

The pressurizing conditions were maintained as in the previous case. However, unlike the previous simulation, the upper part containing the hole was positioned with varying gaps based on different design parameters. The closing pressure was identified when the contact force reaches 0.001 N (see the “Geometric scalability through a model-based scaling design methodology” section).

### Estimation of flow resistance from FEA-deformed channel geometry

The deformation-dependent hydraulic resistance of the curved channel was estimated from the nodal coordinates exported from FEA analysis. For each deformation frame, the nodes on the two opposing channel walls were exported separately and used to reconstruct the local channel geometry. For each wall, nodes with similar in-plane coordinates were grouped into local wall columns. The center position of each column was used to define the wall center curve, while the spread of nodes along the vertical direction was used to estimate the local channel height. The wall center curves were then ordered along the channel path and resampled by arc length using 500 sampling points. The two wall curves were aligned to ensure that they followed the same direction along the channel. A channel midline was calculated as the midpoint between the two resampled wall curves. At each point along this midline, a normal slice was constructed perpendicular to the local channel tangent. The intersections of this normal slice with the two wall boundaries were used to calculate the local channel width. The local channel height was calculated as the average height estimated from the two opposing walls. The channel was then divided into short segments along the midline, where each segment had length, width, and height. Each segment was approximated as a locally rectangular channel. Assuming laminar pressure–driven flow, the hydraulic resistance of each segment was calculated. The total channel resistance was obtained by summing the resistance contributions of all segments along the reconstructed channel path. Because the calculation only uses the reconstructed channel geometry, it does not include additional experimental losses from tubing, connectors, or inlet/outlet regions.

### Experiment comparing channel resistance to external force

This section describes the experimental procedure used to evaluate the resistance of the channels to external loading by measuring the force required to reach an equivalent flow resistance. The same setup used for relative flow resistance measurements was used. As the applied force increases, the channel deforms and its flow resistance increases. When channel flow resistance is low, the inlet pressure $P_{\text{inlet}}$ remains low because most of the pressure drop occurs across the constant reference resistance $R_{\text{ref}}$, which is larger than the channel resistance. However, as channel flow resistance increases, the resistance ratio between the channel and $R_{\text{ref}}$ increases, causing $P_{\text{inlet}}$ to rise. We quantified the external force required to reach a target inlet pressure $P_{\text{inlet}}$ = 45 kPa under a fixed source pressure of $P_{\text{source}}$ = 50 kPa. A load cell (JLC-M1KN, JISC Ltd., Japan) connected to an indenter (universal load tester, HIT, Japan) was used to measure the applied force under displacement-controlled loading. The indenter was positioned at the center of the channel to ensure consistent loading conditions.

## Supplementary Materials

### The PDF file includes:

Notes S1 to S6 Figs. S1 to S8 Tables S1 to S11 Legend for movie S1 References

## References

[1] G. M. Whitesides, The origins and the future of microfluidics. Nature 442, 368–373 (2006). 10.1038/nature05058 16871203

[2] Q. Lu, H. Xu, Y. Guo, J. Y. Wang, L. Yao, “Fluidic computation kit: Towards electronic-free shape-changing interfaces,” in *Proceedings of the 2023 CHI Conference on Human Factors in Computing Systems* (Association for Computing Machinery, 2023), pp. 1–21.

[3] A. P. Singh, M. Tintelott, E. Moussavi, S. Ingebrandt, R. Leupers, X.-T. Vu, F. Merchant, V. Pachauri, Logic operations in fluidics as foundation for embedded biohybrid computation. Device 1, 100220 (2023).

[4] A. A. Stanley, E. S. Roby, S. J. Keller, High-speed fluidic processing circuits for dynamic control of haptic and robotic systems. Sci. Adv. 10, eadl3014 (2024). 10.1126/sciadv.adl3014 PMC10990265 38569043

[5] M. Rhee, M. A. Burns, Microfluidic pneumatic logic circuits and digital pneumatic microprocessors for integrated microfluidic systems. Lab Chip 9, 3131–3143 (2009). 10.1039/b904354c PMC2917228 19823730

[6] H. Lin, J. Tan, J. Zhu, S. Lin, Y. Zhao, W. Yu, H. Hojaiji, B. Wang, S. Yang, X. Cheng, Z. Wang, E. Tang, C. Yeung, S. Emaminejad, A programmable epidermal microfluidic valving system for wearable biofluid management and contextual biomarker analysis. Nat. Commun. 11, 4405 (2020). 10.1038/s41467-020-18238-6 PMC7467936 32879320

[7] R. Z. Gao, C. L. Ren, Synergizing microfluidics with soft robotics: A perspective on miniaturization and future directions. Biomicrofluidics 15, 011302 (2021). 10.1063/5.0036991 PMC7861881 33564346

[8] J. D. Hubbard, R. Acevedo, K. M. Edwards, A. T. Alsharhan, Z. Wen, J. Landry, K. Wang, S. Schaffer, R. D. Sochol, Fully 3D-printed soft robots with integrated fluidic circuitry. Sci. Adv. 7, eabe5257 (2021). 10.1126/sciadv.abe5257 PMC8279518 34261646

[9] S. Song, S. Joshi, J. Paik, CMOS-inspired complementary fluidic circuits for soft robots. Adv. Sci. 8, 2100924 (2021). 10.1002/advs.202100924 PMC8529426 34459157

[10] P. Rothemund, A. Ainla, L. Belding, D. J. Preston, S. Kurihara, Z. Suo, G. M. Whitesides, A soft, bistable valve for autonomous control of soft actuators. Sci. Robot. 3, eaar7986 (2018). 10.1126/scirobotics.aar7986 33141749

[11] Y. Matia, G. H. Kaiser, R. F. Shepherd, A. D. Gat, N. Lazarus, K. H. Petersen, Harnessing nonuniform pressure distributions in soft robotic actuators. Adv. Intell. Syst. 5, 2200330 (2023).

[12] L. C. van Laake, J. de Vries, S. M. Kani, J. T. Overvelde, A fluidic relaxation oscillator for reprogrammable sequential actuation in soft robots. Matter 5, 2898–2917 (2022).

[13] S. Wang, L. He, Y. Yao, C. Liu, P. Maiolino, A toolbox for designing 3D-printing-ready pneumatic circuits for controlling soft robots. Adv. Intell. Syst. 5, 2300394 (2023).

[14] N. W. Bartlett, K. P. Becker, R. J. Wood, A fluidic demultiplexer for controlling large arrays of soft actuators. Soft Matter 16, 5871–5877 (2020). 10.1039/c9sm02502b 32249863

[15] D. Drotman, S. Jadhav, D. Sharp, C. Chan, M. T. Tolley, Electronics-free pneumatic circuits for controlling soft-legged robots. Sci. Robot. 6, eaay2627 (2021). 10.1126/scirobotics.aay2627 34043527

[16] N. Vasios, A. J. Gross, S. Soifer, J. T. B. Overvelde, K. Bertoldi, Harnessing viscous flow to simplify the actuation of fluidic soft robots. Soft Rob. 7, 1–9 (2020). 10.1089/soro.2018.0149 31070518

[17] E. Gallardo Hevia, C. M. McCann, M. Bell, N. P. Hyun, C. Majidi, K. Bertoldi, R. J. Wood, High-gain microfluidic amplifiers: The bridge between microfluidic controllers and fluidic soft actuators. Adv. Intell. Syst. 4, 2200122 (2022).

[18] D. J. Preston, P. Rothemund, H. J. Jiang, M. P. Nemitz, J. Rawson, Z. Suo, G. M. Whitesides, Digital logic for soft devices. Proc. Natl. Acad. Sci. U.S.A. 116, 7750–7759 (2019). 10.1073/pnas.1820672116 PMC6475414 30923120

[19] V. T. Vo, A. Rajappan, B. Jumet, M. D. Bell, S. Urbina, D. J. Preston, Sheet-based fluidic diodes for embedded fluidic circuitry in soft devices. Adv. Intell. Syst. 6, 2300785 (2024).

[20] S. Conrad, J. Teichmann, P. Auth, N. Knorr, K. Ulrich, D. Bellin, T. Speck, F. J. Tauber, 3D-printed digital pneumatic logic for the control of soft robotic actuators. Sci. Robot. 9, eadh4060 (2024). 10.1126/scirobotics.adh4060 38295189

[21] S. Picella, C. M. van Riet, J. T. Overvelde, Pneumatic coding blocks enable programmability of electronics-free fluidic soft robots. Sci. Adv. 10, eadr2433 (2024). 10.1126/sciadv.adr2433 PMC11661450 39705364

[22] V. G. Kortman, B. Mazzolai, A. Sakes, J. Jovanova, Perspectives on intelligence in soft robotics. Adv. Intell. Syst. 7, 2400294 (2025).

[23] C. B. Teeple, B. Aktaş, M. C. Yuen, G. R. Kim, R. D. Howe, R. J. Wood, Controlling palm-object interactions via friction for enhanced in-hand manipulation. IEEE Robot. Autom. Lett. 7, 2258–2265 (2022).

[24] M. S. Xavier, C. D. Tawk, A. Zolfagharian, J. Pinskier, D. Howard, T. Young, J. Lai, S. M. Harrison, Y. K. Yong, M. Bodaghi, A. J. Fleming, Soft pneumatic actuators: A review of design, fabrication, modeling, sensing, control and applications. IEEE Access 10, 59442–59485 (2022).

[25] J. Wang, A. Chortos, Control strategies for soft robot systems. Adv. Intell. Syst. 4, 2100165 (2022).

[26] F. Stella, J. Hughes, The science of soft robot design: A review of motivations, methods and enabling technologies. Front. Rob. AI 9, 1059026 (2023). 10.3389/frobt.2022.1059026 PMC9889359 36743292

[27] S. Jadhav, P. E. Glick, M. Ishida, C. Chan, I. Adibnazari, J. P. Schulze, N. Gravish, M. T. Tolley, Scalable fluidic matrix circuits for controlling large arrays of individually addressable actuators. Adv. Intell. Syst. 5, 2300011 (2023).

[28] J. Xu, H. Nguyen, S. H. Jeong, K. Hjort, Miniaturized soft pneumatic actuator matrix with multiplexing control. Adv. Robot. Res. 1, 2400015 (2025).

[29] A. Rajappan, B. Jumet, R. A. Shveda, C. J. Decker, Z. Liu, T. F. Yap, V. Sanchez, D. J. Preston, Logic-enabled textiles. Proc. Natl. Acad. Sci. U.S.A. 119, e2202118119 (2022). 10.1073/pnas.2202118119 PMC9436326 35994641

[30] J. K. Choe, J. Kim, H. Song, J. Bae, J. Kim, A soft, self-sensing tensile valve for perceptive soft robots. Nat. Commun. 14, 3942 (2023). 10.1038/s41467-023-39691-z PMC10319868 37402707

[31] C. J. Decker, H. J. Jiang, M. P. Nemitz, S. E. Root, A. Rajappan, J. T. Alvarez, J. Tracz, L. Wille, D. J. Preston, G. M. Whitesides, Programmable soft valves for digital and analog control. Proc. Natl. Acad. Sci. U.S.A. 119, e2205922119 (2022). 10.1073/pnas.2205922119 PMC9546565 36161907

[32] D. W. Lee, Y. H. Cho, High-radix microfluidic multiplexer with pressure valves of different thresholds. Lab Chip 9, 1681–1686 (2009). 10.1039/b820582e 19495450

[33] D. W. Lee, I. Doh, Y. Kim, Y. H. Cho, Advanced combinational microfluidic multiplexer using multiple levels of control pressures. Lab Chip 13, 3658–3662 (2013). 10.1039/c3lc50513h 23896765

[34] B. V. Raemdonck, E. Milana, M. D. Volder, D. Reynaerts, B. Gorissen, Nonlinear inflatable actuators for distributed control in soft robots. Adv. Mater. 35, e2301487 (2023). 10.1002/adma.202301487 37205727

[35] K. McDonald, T. Ranzani, Hardware methods for onboard control of fluidically actuated soft robots. Front. Rob. AI 8, 720702 (2021). 10.3389/frobt.2021.720702 PMC8415906 34485392

[36] M. A. Robertson, J. Paik, New soft robots really suck: Vacuum-powered systems empower diverse capabilities. Sci. Robot. 2, eaan6357 (2017). 10.1126/scirobotics.aan6357 33157853

[37] A. Shtarbanov, “FlowIO development platform—The pneumatic ‘raspberry pi’ for soft robotics,” in *Extended Abstracts of the 2021 CHI Conference on Human Factors in Computing Systems* (Association for Computing Machinery, 2021), pp. 1–6; 10.1145/3411763.3451513)CHI.

[38] S. Vogel, *Life in Moving Fluids: The Physical Biology of Flow* (Princeton Univ. Press, 1996).

[39] S. Nagels, W. Deferme, Fabrication approaches to interconnect based devices for stretchable electronics: A review. Materials 11, 375 (2018). 10.3390/ma11030375 PMC5872954 29510497

[40] J. P. Rojas, D. Singh, S. B. Inayat, G. A. T. Sevilla, H. M. Fahad, M. M. Hussain, Review—Micro and nano-engineering enabled new generation of thermoelectric generator devices and applications. ECS J. Solid State Sci. Technol. 6, N3036 (2017).

[41] H. Ramachandraiah, S. Ardabili, A. M. Faridi, J. Gantelius, J. M. Kowalewski, G. Mårtensson, A. Russom, Dean flow-coupled inertial focusing in curved channels. Biomicrofluidics 8, 034117 (2014). 10.1063/1.4884306 PMC4162445 25379077

[42] Y. Ying, Y. Lin, Inertial focusing and separation of particles in similar curved channels. Sci. Rep. 9, 1–12 (2019). 10.1038/s41598-019-52983-z PMC6851121 31719582

[43] H. Jeong, J. Feng, J. Kim, 2.5D laser-cutting-based customized fabrication of long-term wearable textile sEMG sensor: From design to intention recognition. IEEE Robot. Autom. Lett. 7, 10367–10374 (2022).

[44] A. A. Amiri Moghadam, S. Alaie, S. Deb Nath, M. Aghasizade Shaarbaf, J. K. Min, S. Dunham, B. Mosadegh, Laser cutting as a rapid method for fabricating thin soft pneumatic actuators and robots. Soft Rob. 5, 443–451 (2018). 10.1089/soro.2017.0069 PMC6094352 29924697

[45] Y. Luo, J. Zou, G. Gu, Multimaterial pneumatic soft actuators and robots through a planar laser cutting and stacking approach. Adv. Intell. Syst. 3, 2000257 (2021).

[46] A. H. McMillan, E. K. Thomée, A. Dellaquila, H. Nassman, T. Segura, S. C. Lesher-Pérez, Rapid fabrication of membrane-integrated thermoplastic elastomer microfluidic devices. Micromachines 11, 731 (2020). 10.3390/mi11080731 PMC7463978 32731570

[47] A. Koivikko, V. Lampinen, M. Pihlajamäki, K. Yiannacou, V. Sharma, V. Sariola, Integrated stretchable pneumatic strain gauges for electronics-free soft robots. Comms. Eng. 1, 14 (2022).

[48] Y. Lian, J. Sun, X. Ge, Z. Yang, X. He, Z. Zheng, A theoretical study of an improved capacitive pressure sensor: Closed-form solution of uniformly loaded annular membranes. Measurement 111, 84–92 (2017).

[49] H. Lin, K. Pan, F. Yang, Q. Qin, “Analysis of stress concentration phenomenon in stretchable interconnects,” in *2015 International Conference on Mechatronics, Electronic, Industrial and Control Engineering (MEIC-15)* (Atlantis Press, 2015), pp. 760–763.

[50] Z. Fan, Y. Zhang, Q. Ma, F. Zhang, H. Fu, K.-C. Hwang, Y. Huang, A finite deformation model of planar serpentine interconnects for stretchable electronics. Int. J. Solids Struct. 91, 46–54 (2016). 10.1016/j.ijsolstr.2016.04.030 PMC5042350 27695135

