# Supplementary material for: Scalable design of fluidic DEMUX/MUX
Source: Sci Adv. 2026 Oct 9;12(41):eaed4468. doi: 10.1126/sciadv.aed4468 (PMC13651841; doi:10.1126/sciadv.aed4468)
Supplement: Supplementary file 1 [file sciadv.aed4468_sm.pdf]

Science Advances
AAAS

Supplementary Materials for

Scalable design of fluidic DEMUX/MUX

Hwayeong Jeong *et al*.

Corresponding author: Jung Kim, jungkim@kaist.ac.kr

Sci. Adv. **12**, eaed4468 (2026)
DOI: 10.1126/sciadv.aed4468

The PDF file includes:

Notes S1 to S6
Figs. S1 to S8
Tables S1 to S11
Legend for movie S1
References

Other Supplementary Material for this manuscript includes the following:

Movie S1

Supplementary Text

Note S1. How to design high-radix demultiplexer (33).

Ternary and quaternary have three and four-state inputs, respectively, but because they also include an input of 0 kPa, there are only two or three pressurized states. Therefore, with m valves that close at different pressures, it is possible to implement $m+1$ states. Let's assume we are determining the maximum number of outputs that can be controlled when constructing a circuit with n control lines using m distinct types of valves. Initially, we shall assume that only the valves designed to close at pressure P_x shall be closed when P_x is applied without considering any interdependencies between the valves. In this scenario, there are $m \times n$ available choices (Table S1), and q of these must be selected to determine the inputs of the control lines.
We also include the option of selecting multiple inputs on a single line, as multiple inputs may be necessary to close valves with different closing pressures. This is because we are currently not considering interdependencies for the sake of design convenience.
The number of possible combinations when selecting y items from a set of x options, regardless of order, is as follows.

$$ {}_xC_y = \frac{x!}{y!\,(x-y)!} \tag{S1} $$

The value of y that results in the maximum number of possible combinations is $y = x/2$. Therefore, returning to the previous problem, when selecting q items from a total of $m \times n$ choices to determine the input, the highest number of possible combinations is achieved when q equals to $(m \times n)\ /\ 2$.
If $[a_1, a_2, \cdots a_m]$ represents the corresponding number of selections for each pressure option, $[P_1, P_2, \cdots P_m]$, the following equation needs to be satisfied.

$$ a_1 + a_2 + \cdots + a_m = \frac{m \times n}{2} \tag{S2} $$

As seen in Table S1, it is important to note that $[a_1, a_2, \cdots a_m]$ must all be less than n. Considering pressure dependency, the equation needs to be modified. If P_x is selected, all pressure values from P_1 to P_{x-1} are also automatically on that selection. Considering this dependency, the previous equation can be modified as follows.

$$ a_1 + 2a_2 + \cdots + ma_m = \frac{m \times n}{2} \tag{S3} $$

Furthermore, due to the introduction of dependency, only one input can be provided per control line. Therefore, the following equation is required to be satisfied.

$$ a_1 + a_2 + \cdots + a_m \leq n \tag{S4} $$

The reason why cases where the sum of quantities is less than n are included, is because there are also states where no input is given (i.e., 0 kPa). Once all possible combinations of $[a_1, a_2, \cdots a_m]$

have been determined in this way, the total number of possible outputs can be calculated as follows.

$$T_{\mathrm{single}} = {}_{n}C_{a_1} \times {}_{n-a_1}C_{a_2} \times {}_{n-a_1-a_2}C_{a_3} \times \cdots \times {}_{n-a_1-a_2-\cdots-a_{m-1}}C_{a_m} \tag{S5}$$

After calculating all possible T_{single} that each combination can create, adding them together yields the maximum number of outputs that can be achieved under that condition.

We now have the necessary information about the required input combinations and need to convert this into a valve arrangement. P_x means that that point remains open when P_x is applied, meaning a valve that closes at one state higher positions at that point. For example, in a ternary system, V_2 is placed at the P_1 points, as applying P_2 causes them to close, while P_2 points remain without valves, staying open at all times.

Note S2. How to determine all possible unique primary patterns.

The method for finding all unique patterns that the designed demultiplexer can generate is as follows. First, we converted the implemented demultiplexer into array of numbers. Assuming we have $m + 1$ states, including 0 kPa, we can convert the valves that close at each pressure into numbers using Table S2. In addition, each input can be also converted to number (Table S3). When we give input 2 (P_2), valve 1 and 0 (Valve closing at P_1 & P_2) closes, and the rest opens. Therefore, we assign output 1 (closed state) if it is smaller than the input and 0 (open state) if it is greater than or equal to the input. After determining the open and closed states of all valves for a specific input, the final selected output for that input is the line where all valves are open, i.e., all valves are in the 0 states. After examining all the possible outputs for each input and extracting only the unique combinations, we can determine all the outputs that the demultiplexer can generate. In case of two ternary inputs, it can generate 7 unique primary patterns with 3 outputs, two quaternary inputs can generate 11 unique primary patterns with 4 outputs.

Note S3. How to determine the minimum input set for the desired output.

Algorithm 1 Selection of Minimum Input Set for Desired Output

---

Input: Primary patterns, Desired output
Output: Minimum input set to achieve the desired output
Initialize pool of valid patterns and inputs: valid patterns ← ∅, valid inputs ← ∅
for each primary pattern in the set of primary patterns **do**
    if the primary pattern has deactivated states in the same positions as the desired output **then**

Add this primary pattern and its corresponding input to the pool of valid patterns and inputs
    end if
end for
Initialize new desired output as the given target desired output
while new desired output still has activated states **do**

Select the pattern from the valid patterns that has the highest number of activated chambers at locations matching the desired output
For each activated chamber in the selected pattern, set the corresponding location in the desired output to inactivated
Set the new desired output as the modified target desired output
end while
Return: The selected input pattern set

Note S4. Diaphragm model.

In the case where the pressure acts uniformly on the diaphragm with the disk, the vertical position of the disk can be expressed as follows (48).

$$w = \left(\frac{a^4 q}{tE}\right)^{1/3} \sum_{i=0}^{\infty} C_i \left(\frac{b-a}{2a}\right)^i, \tag{S6}$$

where E is Young's modulus of the material and the C_i is the constant determined from the Poisson's ratio of the material, ν and the b/a. Therefore, the C_i can be expressed as follows.

$$C_i = f\left(\nu, \frac{b}{a}\right) \tag{S7}$$

To maintain the performance of the valve, it is necessary to achieve a closed state under the same pressure. Therefore, we rearrange the equation for q, the pressure.

$$q = \frac{w^3 tE}{a^4} \frac{1}{\sum_{i=0}^{\infty} C_i \left(\frac{b-a}{2a}\right)^i} \tag{S8}$$

Hence, C_i is a function of ν and b/a, normalizing the pressure by the characteristic pressure scale $w^3 tE/a^4$, the governing relationship can be written in dimensionless form a

$$q \frac{a^4}{w^3 tE} = f\left(\nu, \frac{b}{a}\right) \tag{S9}$$

Note S5. How to measure bonding strength.

A delamination test was conducted to determine the burst pressure by fabricating samples in the shape of a chamber with a membrane spanning across it, as shown in fig. S5A.
Therefore, air injection into one side of the sample generates no airflow from the other side due to the membrane. However, as the pressure increases and the membrane ruptures, pressure and airflow are detected from the outlet side. For the experiment, the membrane thickness was initially set to 1.2 mm and five samples were fabricated and tested. We incrementally increased the pressure by 10 kPa and observed the pressure at which the sample ruptured and the location of the rupture.
TPE showed an average burst pressure of 154 kPa and a standard deviation of 30 kPa (table S8). To investigate how much the TPE-TPE bonding area can be minimized, samples were created by varying the thickness of the chamber separation film. Four different thicknesses [0.6 mm, 0.8 mm, 1.0 mm, 1.2 mm] were used for this test. The results showed that at 1.2 mm, the connector was detached from the membrane first due to channel expansion (the connector is rigid), and the remaining ones burst at the intermediate membrane at the pressure shown in table S9. Based on the results, a bonding width greater than 0.8 mm can withstand pressures exceeding 100 kPa. However, in the case of the delamination sample, when the inter-layer is attached to a wide plane, the bonding can be relatively stable. However, when it is designed as a channel (fig. S5B), one side is completely cut, and the attachment area decreases, leading to relatively unstable bonding. Ideally, there is no difference between the two sides, but in reality, this decrease in contact area occurs because the cutting is not perfectly straight and results in an angled cut (fig. S4C). When TPE is completely cut, a relatively large area is lost during laser cutting, and during heat pressing, there may be mis-location due to a slightly uneven pressure distribution, which can result in a decrease in the bonding area. Therefore, we aim to investigate the burst pressure in channel-structured designs. Initially, we excluded the 0.6 mm sample, which exhibited low burst pressure, and proceeded to create a total of four samples, increasing from 0.8 mm with a 0.4 mm interval.
As a result, in the case of 0.8 mm, the entire side was cut off to form the channel, resulting in insufficient contact area and making it difficult for layer stacking. Therefore, only three samples were tested to obtain their burst pressure. Considering that in the previous experiments, we observed connector detachment at pressures exceeding 200 kPa, the observation range was limited to 180 kPa. As a result, the repaired 1.2 mm sample experienced a burst at a location other than the repaired area at 140 kPa, while the other two samples did not experience a burst up to 180 kPa (table S10).

Note S6. How to design horseshoe structure.

The design of the horseshoe structure involves several parameters, such as the width of the channel, the angle and radius of the curve, and the length of the arm (table S11) (41, 42, 49, 50), as illustrated in fig. S7A. Unlike electrical connectors, pneumatic channels require a wider channel to allow for flow and a thicker wall to minimize the effect of pressurization during actuation.

Condition 1: The structure should not overlap each other.
The most fundamental design rule is that the structures should not overlap, which can be expressed with the following equation.

$$a - b \geq \frac{1}{2}\left(R + \frac{W}{2}\right), \tag{S10}$$

where

$$a = R\cos\left(\theta - \frac{\pi}{2}\right) \tag{S11}$$

$$b = \frac{L}{2}\cos(\pi - \theta) \tag{S12}$$

$$R\cos\left(\theta - \frac{\pi}{2}\right) - \frac{L}{2}\cos(\pi - \theta) \geq \frac{1}{2}\left(R + \frac{W}{2}\right) \tag{S13}$$

Case 1. if $L = 0$

$$R\cos\left(\theta - \frac{\pi}{2}\right) \geq \frac{1}{2}\left(R + \frac{W}{2}\right) \tag{S14}$$

$$R\sin\theta \geq \frac{1}{2}\left(R + \frac{W}{2}\right) \tag{S15}$$

$$\sin\theta \geq \frac{1}{2}\left(1 + \frac{W}{2R}\right) \tag{S16}$$

Case 2. if $L \neq 0$

$$R\cos\left(\theta - \frac{\pi}{2}\right) - \frac{L}{2}\cos(\pi - \theta) \geq \frac{1}{2}\left(R + \frac{W}{2}\right) \tag{S17}$$

$$R\sin\theta + \frac{L}{2}\cos\theta \geq \frac{1}{2}\left(R + \frac{W}{2}\right) \tag{S18}$$

$$\sin\theta + \frac{L}{2R}\cos\theta \geq \frac{1}{2}\left(1 + \frac{W}{2R}\right) \tag{S19}$$

In another expression, the distance between the parts located close to each other, that is, the length of the space on the line connecting the centers of two curves, must be greater than 0. It can be described with the following equation,

$$(a-b)-\left(R+\frac{W}{2}\right)+a-b=2a-2b-\left(R+\frac{W}{2}\right) \tag{S20}$$

$$2R\sin\theta+L\cos\theta-\left(R+\frac{W}{2}\right)>0 \tag{S21}$$

For convenience, we transformed θ to $\gamma+90°$, ensuring that the both $\sin\gamma,\ \cos\gamma$ has positive values. The revised equation is as follows.

$$2R\cos\gamma-L\sin\gamma-\left(R+\frac{W}{2}\right)>0 \tag{S22}$$

In this case, the maximum value of γ that satisfies this equation occurs when the result of the equation is 0, indicating that the structures are in direct contact. To determine the maximum value of γ, we solve the equation to find the γ value that makes the equation result in 0.

$$\sin\gamma=\frac{2\tan\frac{\gamma}{2}}{1+\tan^2\frac{\gamma}{2}},\cos\gamma=\frac{1-\tan^2\frac{\gamma}{2}}{1+\tan^2\frac{\gamma}{2}} \tag{S23}$$

$$\tan\frac{\gamma}{2}=\frac{-2L}{6R+W}+\sqrt{\frac{4L^2+12R^2-4RW-W^2}{(6R+W)^2}} \tag{S24}$$

$$\gamma=2\arctan\left(\frac{-2L}{6R+W}+\sqrt{\frac{4L^2+12R^2-4RW-W^2}{(6R+W)^2}}\right) \tag{S25}$$

A second design guideline is taken from dimensional constraints in external conditions.

Condition 2. Vertical length should meet the external design constraint.
The proposed pneumatic circuit is intended to be attached to the soft actuator later, and the usable area is determined according to the size of the target actuator. Therefore, the limitation in the vertical length should be taken into consideration when choosing the design parameters. To achieve a compact design, keeping the vertical length lower than the valve diameter is better. The vertical length can be defined as follows.

$$Y\leq W+2R-2R\cos\theta+L\sin\theta \tag{S26}$$

Same as in condition 1, it is advisable to identify the condition where the equality is satisfied to determine the maximum value.

Condition 3. Minimize $\epsilon_{\mathrm{crest}}/\epsilon_{\mathrm{eff}}$ to obtain high stretchability.
In the horseshoe design, the crest region experiences the highest local strain during stretching. Therefore, we used the dimensionless ratio to compare stretchability across different geometries

$$\frac{\epsilon_{\mathrm{crest}}}{\epsilon_{\mathrm{eff}}}, \qquad (S27)$$

where $\epsilon_{\mathrm{crest}}$ is the maximum strain at the crest and ϵ_{eff} is the effective applied strain of the overall structure. A lower value of $\epsilon_{\mathrm{crest}}/\epsilon_{\mathrm{eff}}$ indicates that the imposed deformation is accommodated with less local strain concentration, corresponding to higher stretchability.

We first performed 2D finite element simulations to compare the effects of the main design parameters. The material was modeled as linear elastic and nearly incompressible, with $E = 2 \times 10^6$ Pa and Poisson's ratio: 0.499. Because the same material model was used for all designs and the comparison was based on the dimensionless ratio $\epsilon_{\mathrm{crest}}/\epsilon_{\mathrm{eff}}$, the simulations were used to identify relative geometric trends rather than absolute material-dependent strain values. The curve radius was fixed at R = 5 mm, and all designs were compared at 50 % effective strain.

The parametric sweep showed that smaller W/R, larger L/R, and larger θ generally reduced $\epsilon_{\mathrm{crest}}/\epsilon_{\mathrm{eff}}$ (fig. S7B-E). These trends indicate that smoother curvature, longer arms, and larger curve angles allow more deformation to be accommodated through rotation and straightening of the horseshoe geometry, thereby reducing local strain concentration at the crest.

Next, we narrowed the design space by enforcing Conditions 1 and 2 simultaneously. This step was required because the final channel must avoid self-overlap while satisfying the available vertical space in the soft-robot body. Based on the bonding-strength results and the flow-rate requirement, the total channel width was fixed at 4 mm. We then identified geometries satisfying both constraints and simulated each design at 100% effective strain.

In this constrained design space, W/R, L/R, and θ are no longer independent. Although increasing L/R reduced $\epsilon_{\mathrm{crest}}/\epsilon_{\mathrm{eff}}$ in the unconstrained sweep, larger L/R was coupled with larger W/R when both geometric constraints were enforced. Under these final constraints, the effect of W/R became dominant: designs with smaller W/R produced lower $\epsilon_{\mathrm{crest}}/\epsilon_{\mathrm{eff}}$, indicating higher stretchability. Therefore, the final horseshoe geometry was selected by prioritizing a smaller W/R while satisfying the non-overlap and vertical-size constraints. Based on this criterion, for a 4 mm channel width and an 18 mm valve diameter, the selected design parameters were W = 4 mm, L = 2 mm, R = 4 mm, and θ = 119°.

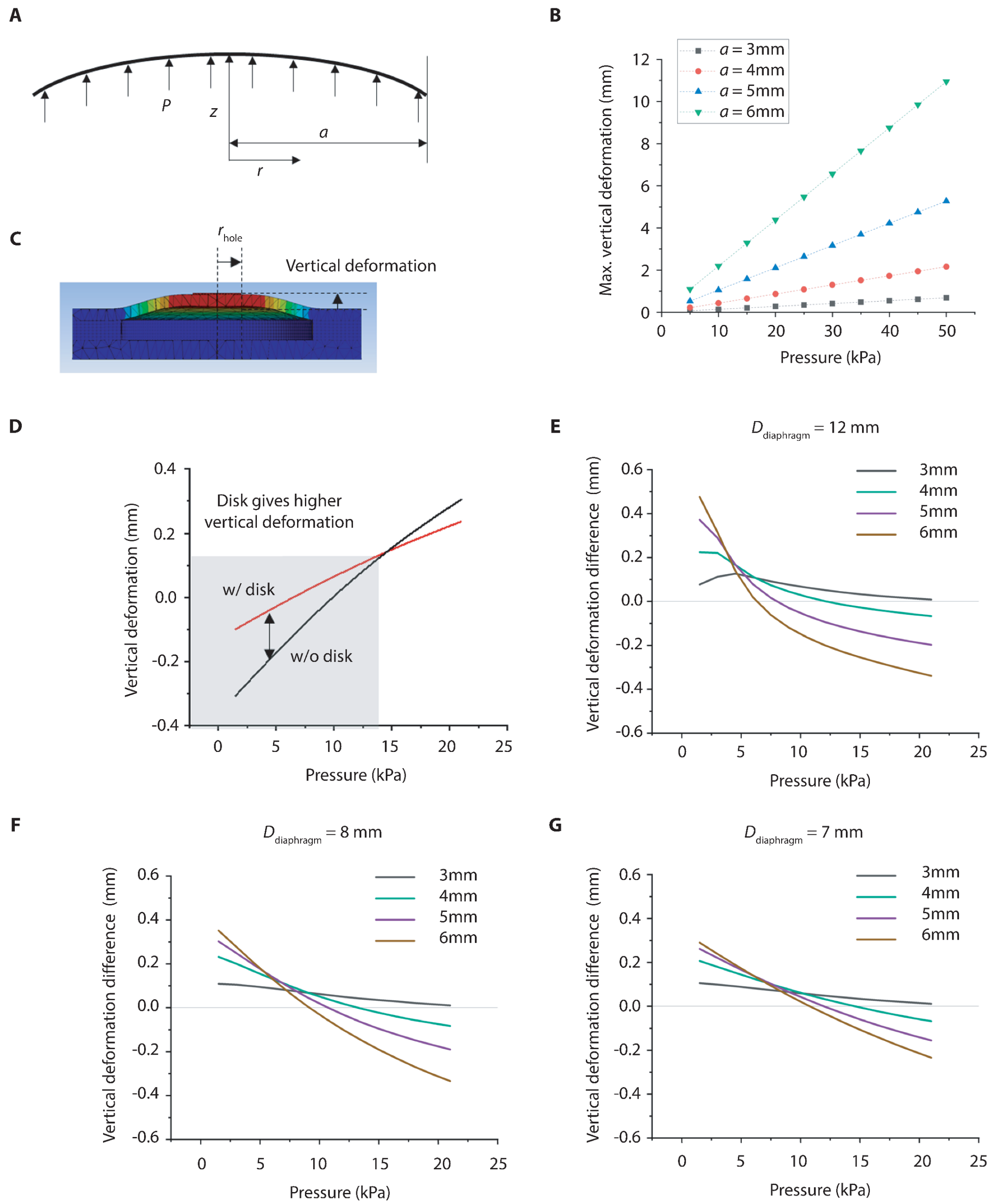

Fig. S1. Analytical model and simulation results used to determine the disk size. (A) Simplified model of a circular diaphragm under uniform pressure. **(B)** Maximum vertical deformation of the diaphragm according to the pressure. **(C)** Location of deformation height extraction in the simulation. **(D)** Condition where the diaphragm with the disk exhibits higher vertical deformation. The difference in deformation between different-sized disks when the diaphragm diameter is **(E)** 12 mm, **(F)** 8 mm, and **(G)** 7 mm.

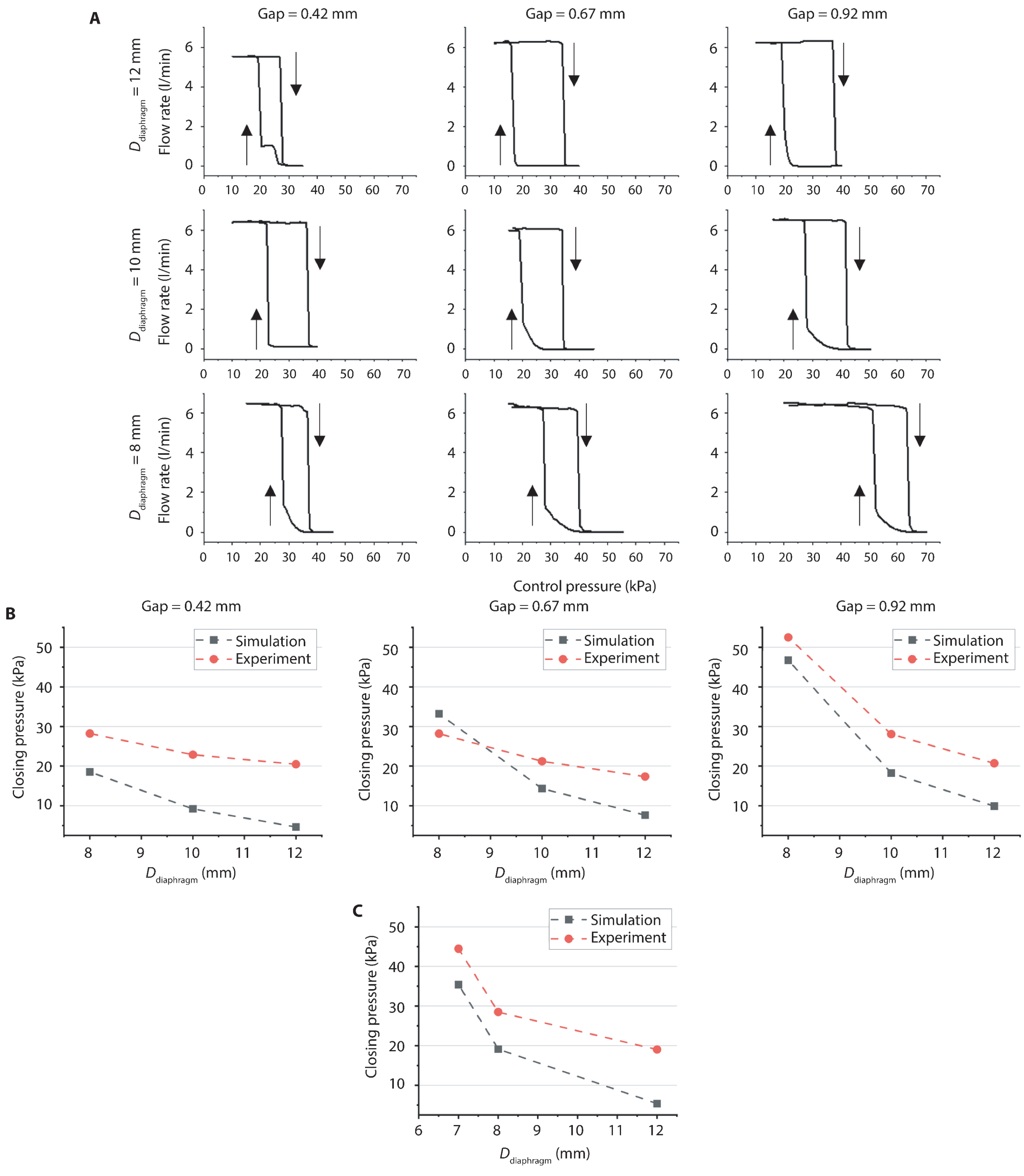

Fig. S2. Experimental and simulation results used to determine the gap and diaphragm diameter. (A) Transfer functions of the valves with varying gap sizes and diaphragm diameters. **(B)** Comparison between experimentally measured and simulated closing pressures for diaphragm diameters of 8, 10, and 12 mm at three different gaps. **(C)** Comparison between experiment and simulation for the selected valve designs. The detailed simulation setup and contact conditions are described in the Materials and Methods section.

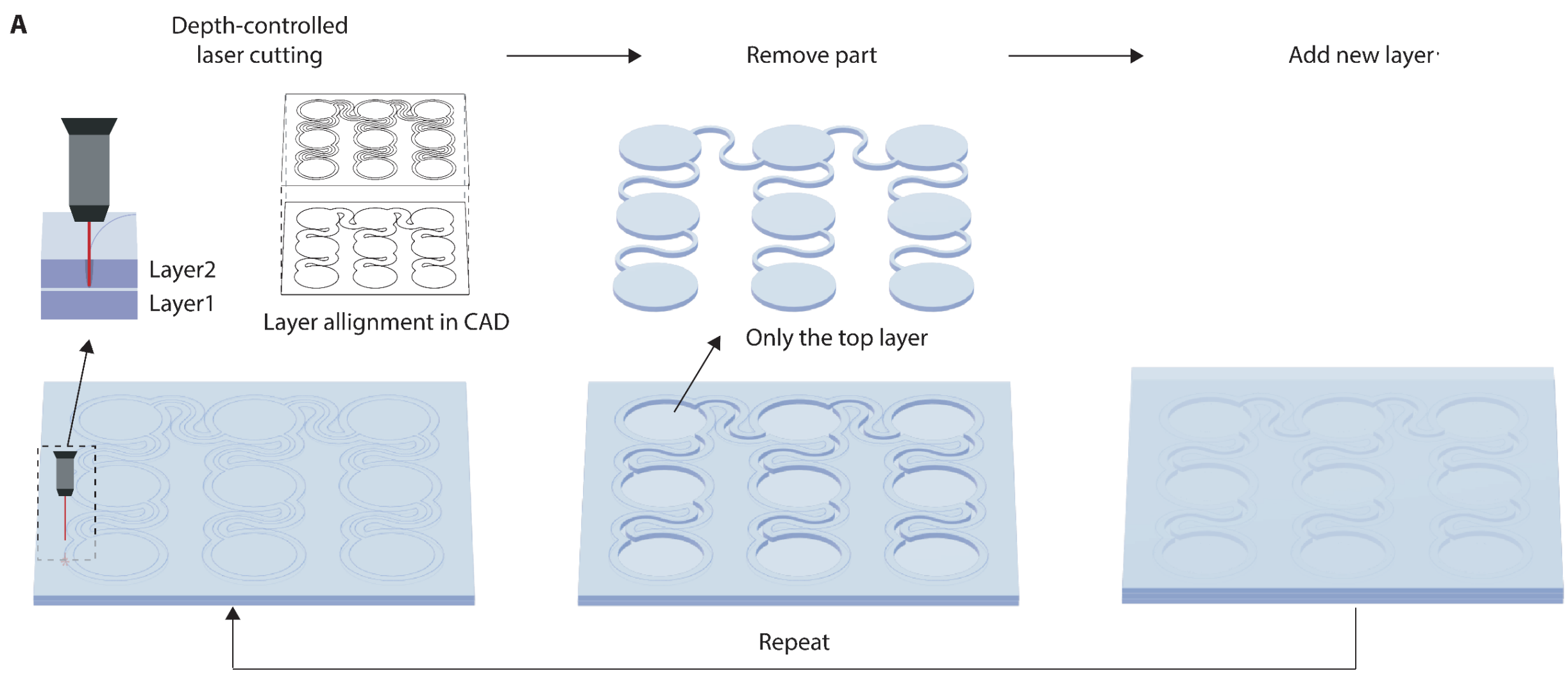

B

Fabricated saparately

	S1	S2	S3
$V_{12\,mm}$	25 kPa	25 kPa	27 kPa
$V_{8\,mm}$	38 kPa	35 kPa	38 kPa
$V_{7\,mm}$	58 kPa	55 kPa	63 kPa

Fig. S3. Scalable 2.5D laser cutting-based additive manufacturing and fabrication repeatability analysis. (A) 2.5D laser cutting-based additive manufacturing technique, enabling scalability in both size and complexity. **(B)** flow-shutoff analysis of three valves, each fabricated individually for their respective designs.

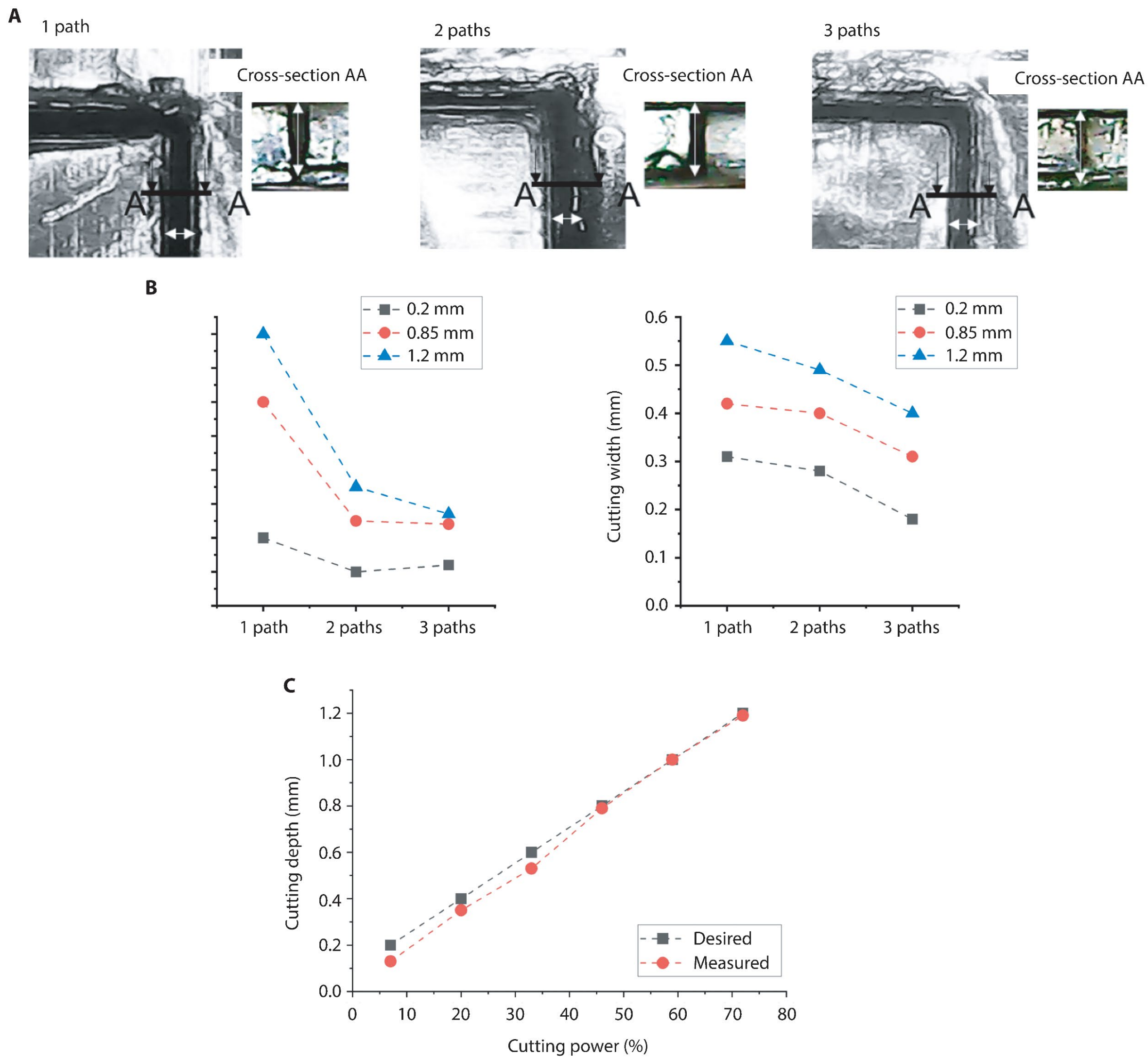

Fig. S4. Effect of laser parameter on cutting depth and width. (A) Results showing the effect of laser power and path on cutting width, capable of cutting 1.2 mm thickness. **(B)** Cutting power and width at a specific penetration depth for different numbers of paths. **(C)** Comparison between desired cutting depth and measured cutting depth.

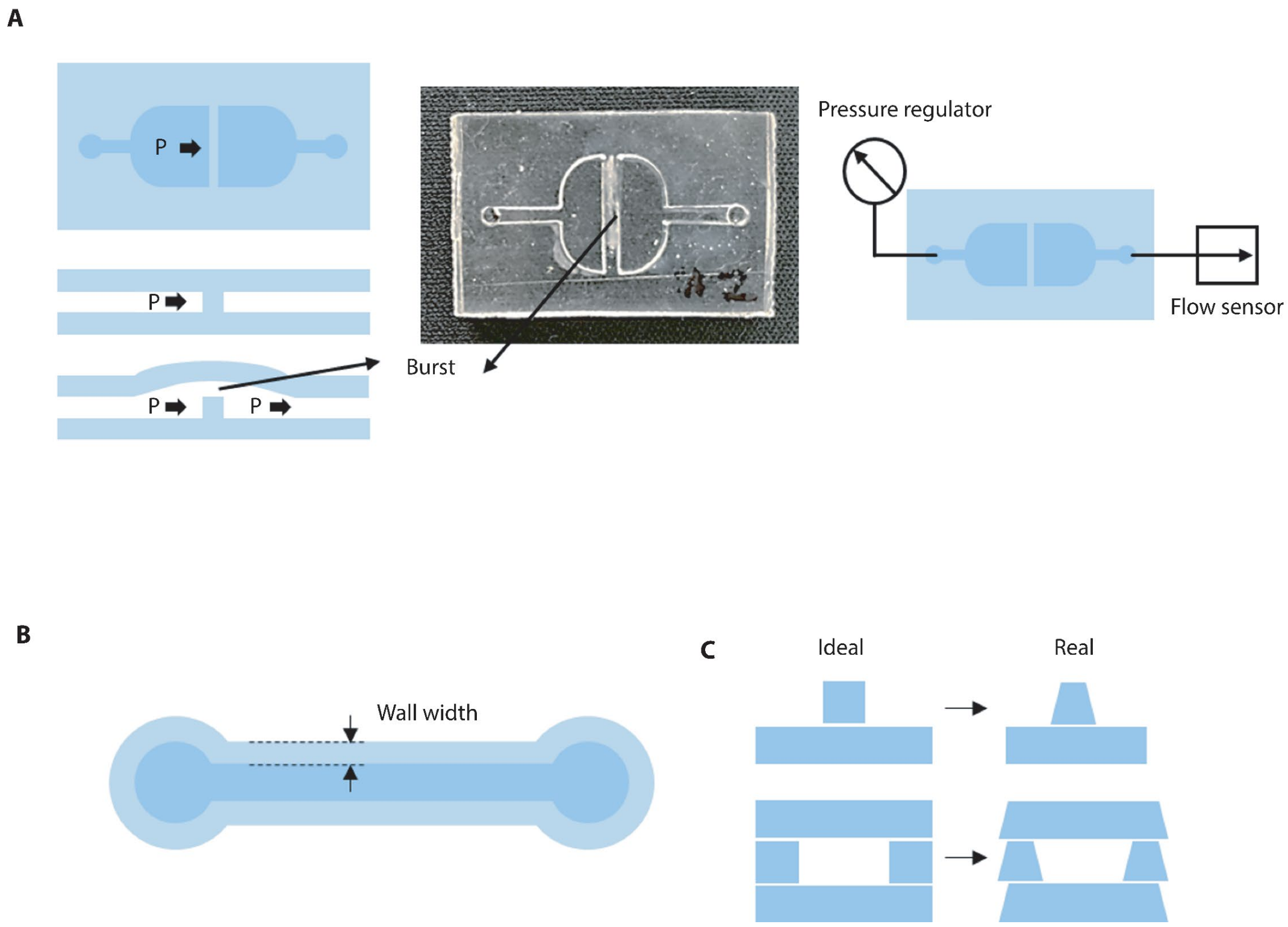

Fig. S5. Bonding strength test. (A) Delamination test specimens and experimental setup. **(B)** Test sample with channel structure and control parameters. **(C)** Comparison of the ideal and actual laser-cut cross-sections.

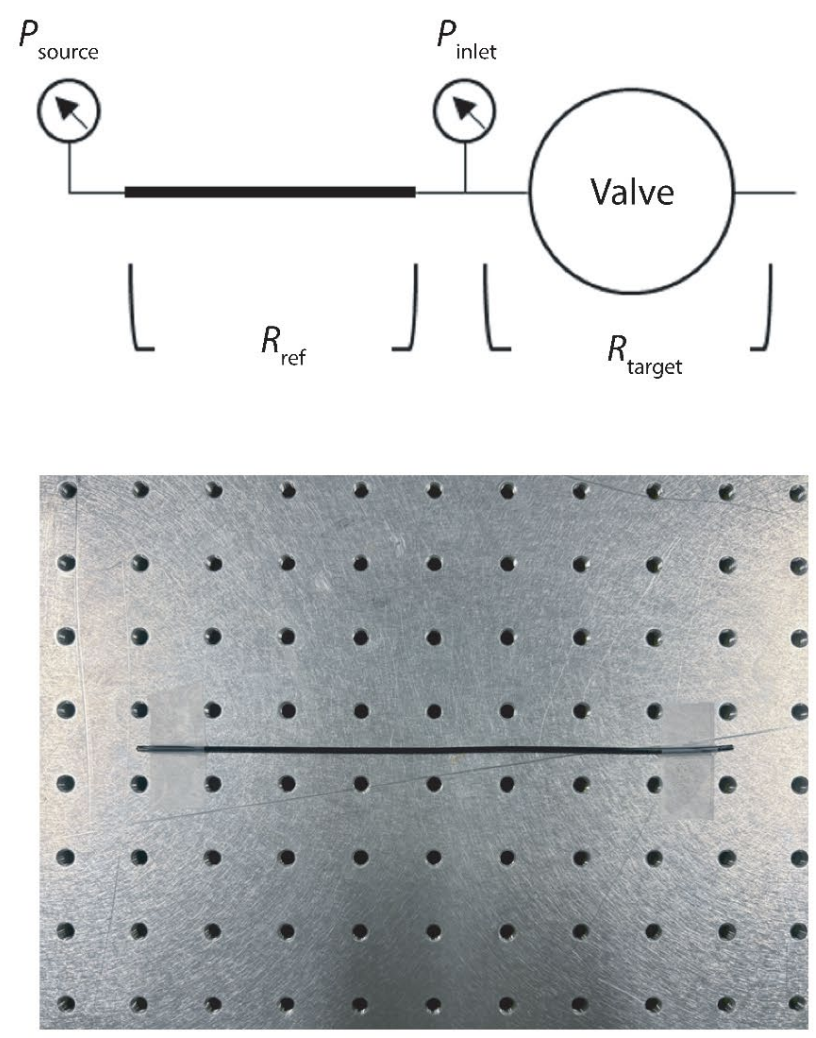

Fig. S6. Experimental setup for measuring the relative flow resistance. A polyurethane tube with a length of 200 mm, an outer diameter of 2 mm, and an inner diameter of 1.2 mm was used as a fixed reference channel.

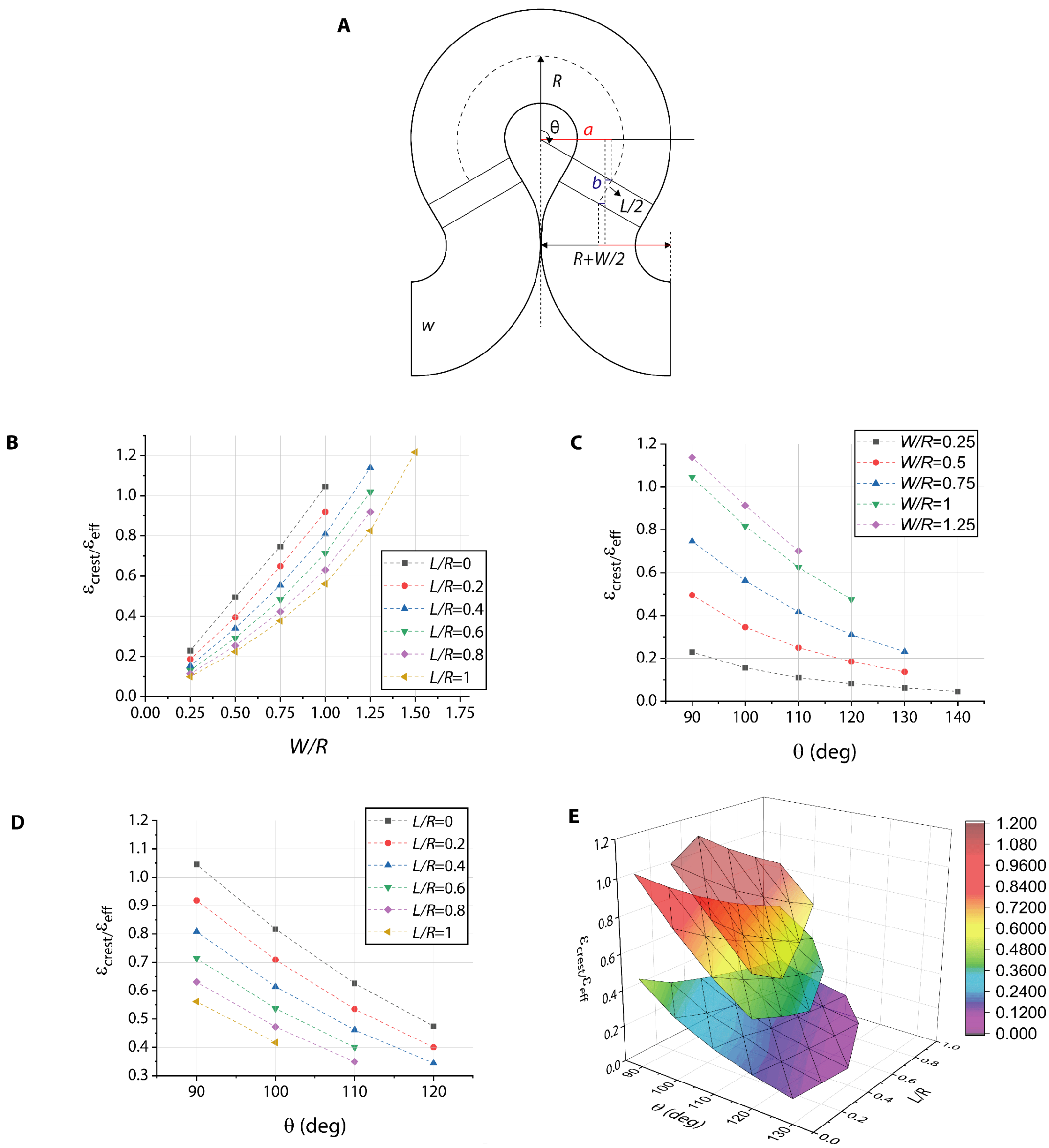

Fig. S7. Horseshoe structure for structural softness and its simulation results on design parameters affecting stretchability. (A) Unit structure of the horseshoe channel in 2D with design parameters. Simulation result of stretchability according to **(B)** $\boldsymbol{W/R}$ for different $\boldsymbol{L/R}$ values, **(C)** $\boldsymbol{\theta}$ for different $\boldsymbol{W/R}$ values, **(D)** $\boldsymbol{\theta}$ for different $\boldsymbol{L/R}$ values, and **(E)** $\boldsymbol{\theta}$ and $\boldsymbol{L/R}$ for different $\boldsymbol{W/R}$ values.

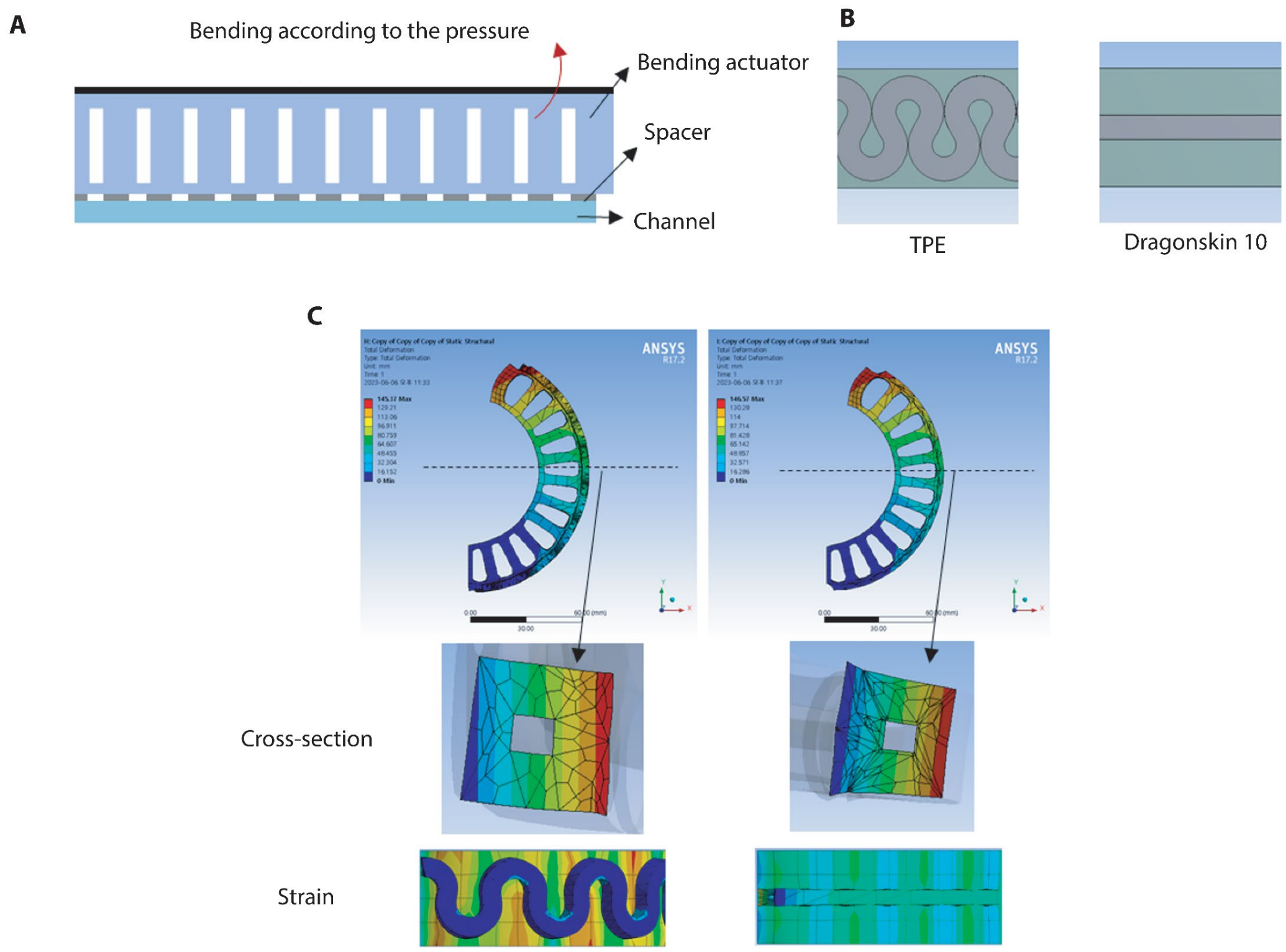

Fig. S8. Simulation setup for testing the integration of the channel on soft robot movement. **(A)** The structure of the channel-integrated bending actuator for the simulation, with a spacer placed between the actuator and the channel to reduce the effects of material differences between the curved channel and the soft robot body. **(B)** The structure of the channels. **(C)** The cross-sectional area of the channel and the strain on the channel when similar bending angles are achieved.

Table S1. All possible pressure input options for n line.

Line 1	P_1	P_2	$\cdots$	P_{m-1}	P_m
Line 2	P_1	P_2	$\cdots$	P_{m-1}	P_m
$\vdots$					
Line $n-1$	P_1	P_2	$\cdots$	P_{m-1}	P_m
Line n	P_1	P_2	$\cdots$	P_{m-1}	P_m

Table S2. A conversion table mapping valves to numbers based on the closing pressure.

Valve closing at P_1	0
Valve closing at P_2	1
Valve closing at P_3	2
No Valve	3

Table S3. A conversion table mapping pressure inputs to numbers.

P_1	1
P_2	2
P_3	3
No input	0

Table S4. Dimensional parameters used for geometric scaling of the valve designs.

Valves in Fig. 4B	Diaphragm thickness (mm)	Disk diameter (mm)	Gap thickness (mm)	Diaphragm diameter (mm)	Hole diameter (mm)
Proportionally scaled-down V_{12mm} valves	0.750	4.00	0.250	12.0	2.00
	0.625	3.33	0.208	10.0	1.67
	0.500	2.67	0.167	8.00	1.33
	0.375	2.00	0.125	6.00	1.00
	0.250	1.33	0.0833	4.00	0.667
	0.125	0.667	0.0417	2.00	0.333

Valves in Fig. 4B	Diaphragm thickness (mm)	Disk diameter (mm)	Gap thickness (mm)	Diaphragm diameter (mm)	Hole diameter (mm)
Proportionally scaled-down V_{8mm} valves	0.75	4	0.25	8	2
	0.5625	3	0.1875	6	1.5
	0.375	2	0.125	4	1
	0.1875	1	0.0625	2	0.5

Valves in Fig. 4B	Diaphragm thickness (mm)	Disk diameter (mm)	Gap thickness (mm)	Diaphragm diameter (mm)	Hole diameter (mm)
Proportionally scaled-down V_{7mm} valves	0.750	4.00	0.250	7.00	2.00
	0.643	3.43	0.214	6.00	1.71
	0.429	2.29	0.143	4.00	1.14
	0.214	1.14	0.0714	2.00	0.571

Valves in Fig. 4C	Diaphragm thickness (mm)	Disk diameter (mm)	Gap thickness (mm)	Diaphragm diameter (mm)	Hole diameter (mm)
Scaled-down V_{12mm} valves with a fixed gap or fixed	0.750	4.00	0.250	12.0	2.00
	0.750	3.33	0.196	10.0	1.67
	0.750	2.67	0.146	8.00	1.33

diaphragm thickness	0.750	2.00	0.0992	6.00	1.00
	0.750	1.33	0.0578	4.00	0.667
	0.362	3.33	0.250	10.0	1.67
	0.148	2.67	0.250	8.00	1.33
	0.0469	2.00	0.250	6.00	1.00
	0.00926	1.33	0.250	4.00	0.667

Valves in Fig. 4D	Diaphragm thickness (mm)	Disk diameter (mm)	Gap thickness (mm)	Diaphragm diameter (mm)	Hole diameter (mm)
V_{12mm} size 1	0.75	4.238	0.27	12.713	2.119
V_{12mm} size 2	1	5.173	0.32	15.518	2.586
V_{12mm} size 3	1.1	5.907	0.37	17.720	2.953
V_{8mm} size 1	0.75	4.238	0.27	8.475	2.119
V_{8mm} size 2	1.12	5.933	0.37	11.867	2.967
V_{8mm} size 3	1.3	6.773	0.42	13.545	3.386
V_{7mm} size 1	0.75	4.238	0.27	7.416	2.119
V_{7mm} size 2	1.28	6.747	0.42	11.806	3.373
V_{7mm} size 3	1.5	8.239	0.52	14.418	4.119

Table S5. Fabrication reproducibility of the V8 size 2 valve closing pressure.

V_{8mm} size 2	Closing pressure (kPa)			
	Trial 1	Trial 2	Trial 3	Mean ± SD
Sample 1	15.02	16.02	16.02	15.69 ± 0.47
Sample 2	14.01	16.01	16.00	15.34 ± 0.94
Sample 3	16.29	16.01	17.02	16.44 ± 0.42

Table S6. Measured cutting depth and width according to the power and the number of paths.

	Power (%)	Cutting depth (mm)	Cutting width (mm)
Path 1	10	Unable to measure	
	20	0.22	0.31
	30	0.45	0.44
	40	0.57	0.42
	50	0.74	0.5
	60	0.83	0.5
	70	0.96	0.53
	80	1.21	0.55
Path 2	10	0.2	0.28
	15	0.35	0.28
	20	0.49	0.34
	25	0.87	0.40
	30	1.00	0.40
	35	1.21	0.49
	40	1.20	0.63
	45	1.28	0.66
Path 3	6	Unable to measure	
	9	Unable to measure	
	12	0.19	0.18
	15	0.34	0.28
	18	0.48	0.28
	21	0.67	0.31
	24	0.82	0.31
	27	1.21	0.40

Table S7. Comparison between desired cutting depth and obtained cutting depth.

Laser power (%)	Desired cutting depth (mm)	Measured cutting depth (mm)
17	0.2	0.13
30	0.4	0.35
43	0.6	0.53
56	0.8	0.79
69	1.0	1.00
82	1.2	1.19

Table S8. Burst pressure of TPE delamination samples.

	Sample 1	Sample 2	Sample 3	Sample 4	Sample 5
Burst pressure (kPa)	125	205	165	125	155
Burst location	Membrane	Connector	Membrane	Membrane	Membrane

Table S9. Burst pressure depending on a thickness of the membrane.

Membrane thickness (mm)	0.6	0.8	1.0	1.2
Burst pressure (kPa)	65	165	135	205
Burst location	Membrane	Membrane	Membrane	Connector

Table S10. The burst pressure test results with channel-structured samples.

Membrane thickness (mm)	0.8	1.2	1.6	2.0
Result	Structurally unstable	140 kPa	Over 180 kPa	Over 180 kPa

Table S11. Design parameters in the horseshoe channel.

Design parameter	Notation
The radius of the curve	R
The angle of the curve	θ
Arm length	L
Width of the channel	W

Movie S1. System-level demonstration of Fluidic DEMUX/MUX

This video demonstrates system-level scalability through single-channel selection and shape rendering, as well as physical integrability validated by actuation under mechanical loading.

REFERENCES

1. G. M. Whitesides, The origins and the future of microfluidics. *Nature* **442**, 368–373 (2006).

2. Q. Lu, H. Xu, Y. Guo, J. Y. Wang, L. Yao, "Fluidic computation kit: Towards electronic-free shape-changing interfaces," in *Proceedings of the 2023 CHI Conference on Human Factors in Computing Systems* (Association for Computing Machinery, 2023), pp. 1–21.

3. A. P. Singh, M. Tintelott, E. Moussavi, S. Ingebrandt, R. Leupers, X.-T. Vu, F. Merchant, V. Pachauri, Logic operations in fluidics as foundation for embedded biohybrid computation. *Device* **1**, 100220 (2023).

4. A. A. Stanley, E. S. Roby, S. J. Keller, High-speed fluidic processing circuits for dynamic control of haptic and robotic systems. *Sci. Adv.* **10**, eadl3014 (2024).

5. M. Rhee, M. A. Burns, Microfluidic pneumatic logic circuits and digital pneumatic microprocessors for integrated microfluidic systems. *Lab Chip* **9**, 3131–3143 (2009).

6. H. Lin, J. Tan, J. Zhu, S. Lin, Y. Zhao, W. Yu, H. Hojaiji, B. Wang, S. Yang, X. Cheng, Z. Wang, E. Tang, C. Yeung, S. Emaminejad, A programmable epidermal microfluidic valving system for wearable biofluid management and contextual biomarker analysis. *Nat. Commun.* **11**, 4405 (2020).

7. R. Z. Gao, C. L. Ren, Synergizing microfluidics with soft robotics: A perspective on miniaturization and future directions. *Biomicrofluidics* **15**, 011302 (2021).

8. J. D. Hubbard, R. Acevedo, K. M. Edwards, A. T. Alsharhan, Z. Wen, J. Landry, K. Wang, S. Schaffer, R. D. Sochol, Fully 3D-printed soft robots with integrated fluidic circuitry. *Sci. Adv.* **7**, eabe5257 (2021).

9. S. Song, S. Joshi, J. Paik, CMOS-inspired complementary fluidic circuits for soft robots. *Adv. Sci.* **8**, 2100924 (2021).

10. P. Rothemund, A. Ainla, L. Belding, D. J. Preston, S. Kurihara, Z. Suo, G. M. Whitesides, A soft, bistable valve for autonomous control of soft actuators. *Sci. Robot.* **3**, eaar7986 (2018).

11. Y. Matia, G. H. Kaiser, R. F. Shepherd, A. D. Gat, N. Lazarus, K. H. Petersen, Harnessing nonuniform pressure distributions in soft robotic actuators. *Adv. Intell. Syst.* **5**, 2200330 (2023).

12. L. C. van Laake, J. de Vries, S. M. Kani, J. T. Overvelde, A fluidic relaxation oscillator for reprogrammable sequential actuation in soft robots. *Matter* **5**, 2898–2917 (2022).

13. S. Wang, L. He, Y. Yao, C. Liu, P. Maiolino, A toolbox for designing 3D-printing-ready pneumatic circuits for controlling soft robots. *Adv. Intell. Syst.* **5**, 2300394 (2023).

14. N. W. Bartlett, K. P. Becker, R. J. Wood, A fluidic demultiplexer for controlling large arrays of soft actuators. *Soft Matter* **16**, 5871–5877 (2020).

15. D. Drotman, S. Jadhav, D. Sharp, C. Chan, M. T. Tolley, Electronics-free pneumatic circuits for controlling soft-legged robots. *Sci. Robot.* **6**, eaay2627 (2021).

16. N. Vasios, A. J. Gross, S. Soifer, J. T. B. Overvelde, K. Bertoldi, Harnessing viscous flow to simplify the actuation of fluidic soft robots. *Soft Rob.* **7**, 1–9 (2020).

17. E. Gallardo Hevia, C. M. McCann, M. Bell, N. P. Hyun, C. Majidi, K. Bertoldi, R. J. Wood, High-gain microfluidic amplifiers: The bridge between microfluidic controllers and fluidic soft actuators. *Adv. Intell. Syst.* **4**, 2200122 (2022).

18. D. J. Preston, P. Rothemund, H. J. Jiang, M. P. Nemitz, J. Rawson, Z. Suo, G. M. Whitesides, Digital logic for soft devices. *Proc. Natl. Acad. Sci. U.S.A.* **116**, 7750–7759 (2019).

19. V. T. Vo, A. Rajappan, B. Jumet, M. D. Bell, S. Urbina, D. J. Preston, Sheet-based fluidic diodes for embedded fluidic circuitry in soft devices. *Adv. Intell. Syst.* **6**, 2300785 (2024).

20. S. Conrad, J. Teichmann, P. Auth, N. Knorr, K. Ulrich, D. Bellin, T. Speck, F. J. Tauber, 3D-printed digital pneumatic logic for the control of soft robotic actuators. *Sci. Robot.* **9**, eadh4060 (2024).

21. S. Picella, C. M. van Riet, J. T. Overvelde, Pneumatic coding blocks enable programmability of electronics-free fluidic soft robots. *Sci. Adv.* **10**, eadr2433 (2024).

22. V. G. Kortman, B. Mazzolai, A. Sakes, J. Jovanova, Perspectives on intelligence in soft robotics. *Adv. Intell. Syst.* **7**, 2400294 (2025).

23. C. B. Teeple, B. Aktaş, M. C. Yuen, G. R. Kim, R. D. Howe, R. J. Wood, Controlling palm-object interactions via friction for enhanced in-hand manipulation. *IEEE Robot. Autom. Lett.* **7**, 2258–2265 (2022).

24. M. S. Xavier, C. D. Tawk, A. Zolfagharian, J. Pinskier, D. Howard, T. Young, J. Lai, S. M. Harrison, Y. K. Yong, M. Bodaghi, A. J. Fleming, Soft pneumatic actuators: A review of design, fabrication, modeling, sensing, control and applications. *IEEE Access* **10**, 59442–59485 (2022).

25. J. Wang, A. Chortos, Control strategies for soft robot systems. *Adv. Intell. Syst.* **4**, 2100165 (2022).

26. F. Stella, J. Hughes, The science of soft robot design: A review of motivations, methods and enabling technologies. *Front. Rob. AI* **9**, 1059026 (2023).

27. S. Jadhav, P. E. Glick, M. Ishida, C. Chan, I. Adibnazari, J. P. Schulze, N. Gravish, M. T. Tolley, Scalable fluidic matrix circuits for controlling large arrays of individually addressable actuators. *Adv. Intell. Syst.* **5**, 2300011 (2023).

28. J. Xu, H. Nguyen, S. H. Jeong, K. Hjort, Miniaturized soft pneumatic actuator matrix with multiplexing control. *Adv. Robot. Res.* **1**, 2400015 (2025).

29. A. Rajappan, B. Jumet, R. A. Shveda, C. J. Decker, Z. Liu, T. F. Yap, V. Sanchez, D. J. Preston, Logic-enabled textiles. *Proc. Natl. Acad. Sci. U.S.A.* **119**, e2202118119 (2022).

30. J. K. Choe, J. Kim, H. Song, J. Bae, J. Kim, A soft, self-sensing tensile valve for perceptive soft robots. *Nat. Commun.* **14**, 3942 (2023).

31. C. J. Decker, H. J. Jiang, M. P. Nemitz, S. E. Root, A. Rajappan, J. T. Alvarez, J. Tracz, L. Wille, D. J. Preston, G. M. Whitesides, Programmable soft valves for digital and analog control. *Proc. Natl. Acad. Sci. U.S.A.* **119**, e2205922119 (2022).

32. D. W. Lee, Y. H. Cho, High-radix microfluidic multiplexer with pressure valves of different thresholds. *Lab Chip* **9**, 1681–1686 (2009).

33. D. W. Lee, I. Doh, Y. Kim, Y. H. Cho, Advanced combinational microfluidic multiplexer using multiple levels of control pressures. *Lab Chip* **13**, 3658–3662 (2013).

34. B. V. Raemdonck, E. Milana, M. D. Volder, D. Reynaerts, B. Gorissen, Nonlinear inflatable actuators for distributed control in soft robots. *Adv. Mater.* **35**, e2301487 (2023).

35. K. McDonald, T. Ranzani, Hardware methods for onboard control of fluidically actuated soft robots. *Front. Rob. AI* **8**, 720702 (2021).

36. M. A. Robertson, J. Paik, New soft robots really suck: Vacuum-powered systems empower diverse capabilities. *Sci. Robot.* **2**, eaan6357 (2017).

37. A. Shtarbanov, "FlowIO development platform—The pneumatic 'raspberry pi' for soft robotics," in *Extended Abstracts of the 2021 CHI Conference on Human Factors in Computing Systems* (Association for Computing Machinery, 2021), pp. 1–6; https://dl.acm.org/doi/10.1145/3411763.3451513.

38. S. Vogel, *Life in Moving Fluids: The Physical Biology of Flow* (Princeton Univ. Press, 1996).

39. S. Nagels, W. Deferme, Fabrication approaches to interconnect based devices for stretchable electronics: A review. *Materials* **11**, 375 (2018).

40. J. P. Rojas, D. Singh, S. B. Inayat, G. A. T. Sevilla, H. M. Fahad, M. M. Hussain, Review—Micro and nano-engineering enabled new generation of thermoelectric generator devices and applications. *ECS J. Solid State Sci. Technol.* **6**, N3036 (2017).

41. H. Ramachandraiah, S. Ardabili, A. M. Faridi, J. Gantelius, J. M. Kowalewski, G. Mårtensson, A. Russom, Dean flow-coupled inertial focusing in curved channels. *Biomicrofluidics* **8**, 034117 (2014).

42. Y. Ying, Y. Lin, Inertial focusing and separation of particles in similar curved channels. *Sci. Rep.* **9**, 1–12 (2019).

43. H. Jeong, J. Feng, J. Kim, 2.5D laser-cutting-based customized fabrication of long-term wearable textile sEMG sensor: From design to intention recognition. *IEEE Robot. Autom. Lett.* **7**, 10367–10374 (2022).

44. A. A. Amiri Moghadam, S. Alaie, S. Deb Nath, M. Aghasizade Shaarbaf, J. K. Min, S. Dunham, B. Mosadegh, Laser cutting as a rapid method for fabricating thin soft pneumatic actuators and robots. *Soft Rob.* **5**, 443–451 (2018).

45. Y. Luo, J. Zou, G. Gu, Multimaterial pneumatic soft actuators and robots through a planar laser cutting and stacking approach. *Adv. Intell. Syst.* **3**, 2000257 (2021).

46. A. H. McMillan, E. K. Thomée, A. Dellaquila, H. Nassman, T. Segura, S. C. Lesher-Pérez, Rapid fabrication of membrane-integrated thermoplastic elastomer microfluidic devices. *Micromachines* **11**, 731 (2020).

47. A. Koivikko, V. Lampinen, M. Pihlajamäki, K. Yiannacou, V. Sharma, V. Sariola, Integrated stretchable pneumatic strain gauges for electronics-free soft robots. *Comms. Eng.* **1**, 14 (2022).

48. Y. Lian, J. Sun, X. Ge, Z. Yang, X. He, Z. Zheng, A theoretical study of an improved capacitive pressure sensor: Closed-form solution of uniformly loaded annular membranes. *Measurement*, **111**, 84–92 (2017).

49. H. Lin, K. Pan, F. Yang, Q. Qin, "Analysis of stress concentration phenomenon in stretchable interconnects," in *2015 International Conference on Mechatronics, Electronic, Industrial and Control Engineering (MEIC-15)* (Atlantis Press, 2015), pp. 760–763.

50. Z. Fan, Y. Zhang, Q. Ma, F. Zhang, H. Fu, K.-C. Hwang, Y. Huang, A finite deformation model of planar serpentine interconnects for stretchable electronics. *Int. J. Solids Struct.* **91**, 46–54 (2016).
